# singIST: an R/bioconductor library and quarto dashboard for automated single-cell comparative transcriptomics analysis of disease models and humans

**DOI:** 10.3389/fgene.2026.1952333

**Published:** 2026-09-25

**Authors:** Aitor Moruno-Cuenca, Sergio Picart-Armada, Alexandre Perera-Lluna, Francesc Fernández-Albert

**Affiliations:** 1 Data Science R&D, Almirall SA, Sant Feliu de Llobregat, Spain; 2 B2SLab, Institut de Recerca i Innovació en Salut (IRIS), Universitat Politècnica de Catalunya, Barcelona, Spain; 3 Networking Biomedical Research Centre in the Subject Area of Bioengineering, Madrid, Spain; 4 Institut de Recerca Sant Joan de Déu, Barcelona, Spain

**Keywords:** data visualization, pathway analysis, R/bioconductor, reproducible research, single-cell transcriptomics, translational bioinformatics

## Abstract

**Background:**

Preclinical disease models frequently fail to recapitulate human pathophysiology at single-cell resolution, complicating model selection and limiting translational relevance. While single-cell RNA sequencing enables detailed characterization of disease mechanisms, systematic and interpretable frameworks to compare disease models against human references across cell types and pathways remain limited. There is a need for standardized, reproducible tools that support quantitative cross-species comparison while remaining accessible to applied researchers.

**Results:**

We present singIST, an R/Bioconductor package for quantitative and interpretable comparison of disease model single-cell transcriptomic data against human reference datasets. singIST integrates pathway-level modeling with cell-type resolution and one-to-one orthology mapping to quantify the direction and magnitude of recapitulation across pathways, cell types, and genes. To facilitate interpretation and reporting, we additionally provide singIST Visualizer, a companion Quarto-based Shiny application enabling interactive exploration of results and automated generation of publication-ready figures and tables without custom code. We demonstrate the workflow using a mouse oxazolone model compared against a human atopic dermatitis reference, assessing biological alignment at multiple levels of resolution.

**Conclusion:**

singIST provides a standardized and reproducible framework for comparative single-cell transcriptomic analysis between disease models and humans, with an emphasis on interpretability and practical usability. By combining quantitative recapitulation metrics with an interactive visualization interface, singIST supports informed model evaluation, hypothesis generation, and translational decision-making in preclinical research. The software is freely available via Bioconductor under an open-source license. The singIST visualizer is freely available in Shiny with 8 GB memory https://amoruno.shinyapps.io/singIST_visualizer/.

## 1 Introduction

Translational biomedical research relies on preclinical disease models that faithfully reproduce human biology at molecular and cellular levels. However, many commonly used models capture only partial aspects of human disease, making model selection a critical yet challenging step with direct implications for interpretation and downstream decision-making in drug discovery. While single-cell RNA sequencing enables detailed characterization of disease mechanisms, quantitative and interpretable frameworks to systematically compare disease models against human references at single-cell resolution remain limited.

Most existing computational approaches for model-to-human comparison focus on bulk transcriptomic measurements and provide summaries that do not resolve cell type–specific effects (Picart-Armada et al., 2024; Normand et al., 2018; Haldar et al., 2025; Almeida-Silva et al., 2025). In the single-cell setting, the most common comparative strategy is instead to compute the overlap of differentially expressed genes (ODEGs) between a disease model and the human condition, often separately for each cell type (Kim et al., 2019; Li et al., 2023; Ali et al., 2024); however, this overlap-based analysis treats every gene’s direction and magnitude as equally important and has been shown to overestimate gene overlap (Lawhorn et al., 2018), motivating approaches that integrate effect direction and magnitude. This limitation is particularly relevant in complex tissues, where disease phenotypes often arise from interactions among distinct cellular compartments. As a result, researchers frequently assemble *ad hoc* workflows involving orthology mapping, pathway definition, model fitting, and result interpretation, leading to analyses that are difficult to standardize, reproduce, and compare across studies.

In addition, comparative single-cell analyses produce high-dimensional outputs across pathways, cell types, and genes, which are challenging to interrogate and communicate using static visualizations alone. This gap between analytical complexity and interpretability limits the practical adoption of advanced comparative methods in applied translational settings.

To address these challenges, we developed *singIST*, an R/Bioconductor package that provides a standardized and reproducible workflow for comparing disease model single-cell transcriptomic data against human reference datasets. singIST enables quantitative and interpretable assessment of biological alignment across pathways, cell types, and genes, and is complemented by *singIST Visualizer*, an interactive application that supports systematic exploration and reporting of results. Together, these tools are designed to facilitate informed evaluation of disease models in translational research workflows.

## 2 Implementation

The methodological framework underlying *singIST* has been previously described and validated as a standalone method (Moruno-Cuenca et al., 2026). In this work, we focus on the software implementation designed to enable its practical use, reproducibility, and standardization in applied translational research settings. *singIST* encapsulates the full analytical workflow into a structured R/Bioconductor package, abstracting methodological complexity while providing consistent inputs, outputs, and interpretation across studies, the visual workflow is shown in Figure 1. To further support accessibility and adoption, the package is complemented by *singIST Visualizer*, an interactive application that enables systematic exploration, comparison, and reporting of results without requiring users to directly manipulate high-dimensional intermediate objects or implement custom visualization code.

**FIGURE 1:**
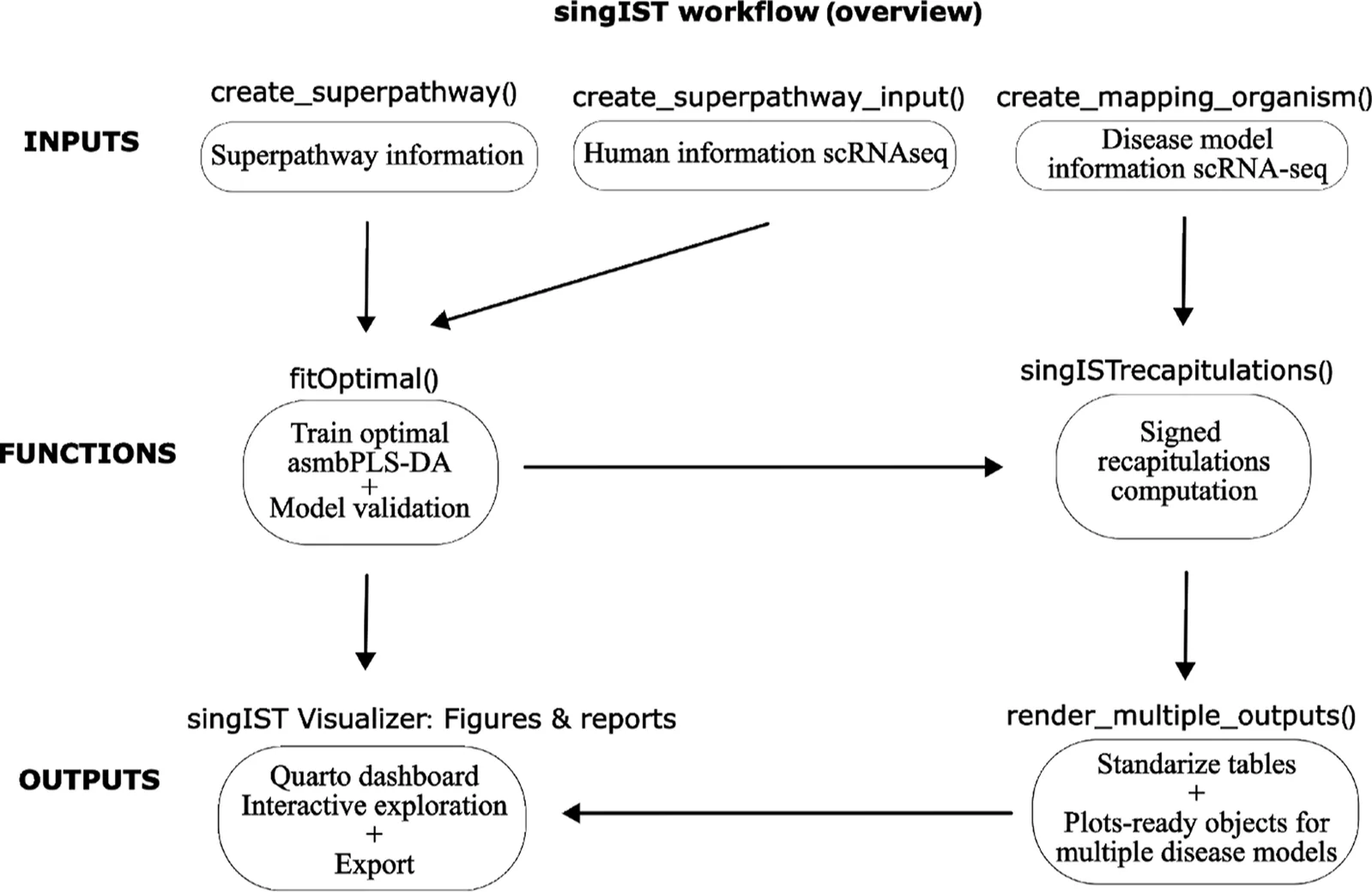
singIST workflow (overview). Input lists (superpathway definition, human reference pseudobulk data with tuning settings, and disease model scRNA-seq with cell type mapping) are created with create_* constructors. fitOptimal() trains and validates the optimal human asmbPLS-DA model, singISTrecapitulations() computes signed recapitulation scores, and render_multiple_outputs() standardizes results into plot-ready tables that can be explored and exported through the Quarto-based singIST Visualizer, which additionally provides a Simulation panel for parameter exploration and benchmarking.

### 2.1 Preparing input data

Before calling any function, users must:

 • Define the superpathways to be analyzed. Each superpathway to be analyzed will be contained in a list created by a dedicated constructor (create_pathway).
 my_pathway <_ create_pathway(standard_name = ”KEGG_CYTOKINE_CYTOKINE_RECEPTOR_INTERACTION”,
 dbsource = ”KEGG”, collection = ”c2”, subcollection = ”CP”)

The create_pathway() constructor supports pathways from databases WIKIPATHWAYS, KEGG, PID, REACTOME and BIOCARTA. Next, the user specifies which cell types to model for that pathway and assigns a gene set to each cell type. There are two options; shared genes: use the same pathway gene set for all chosen cell types; cell type specific genes: supply a distinct gene set for each cell type. If the user chooses cell type specific genes, one must manually supply a list mapping each cell type to its own gene vector. We exemplify the case of shared genes for all cell types.

my_superpathway <_create_superpathway (pathway_info = my_pathway,
celltypes = c(“T-cell”, “Dendritic Cells”))
# Fetch and assign gene sets from MsigDB
# for each modeled cell type
gse <_msigdb::getMsigdb(org = “hs”, id = c(“SYM”, “EZID”),
version = msigdb::getMsigdbVersions() [ 1 ])
my_superpathway <_ setGeneSetsCelltype(my_superpathway, gse = gse)
• Make sure that the human reference data are appropriately pseudobulked and log-normalized. singIST expects a pseudobulk log-normalized matrix aggregated by sample and cell type (rows are sample × celltype profiles; columns are genes). singIST supports generating pseudobulk from either Seurat or SingleCellExperiment workflows (e.g., Seurat::AggregateExpression() for Seurat).
pseudobulk_lognorm <_Seurat::Aggregate Expression (human,
group.by = c(“celltype”, “sampleID”), normalization. method = “LogNormalize”) $RNA$ data
   otherwise if the object is SingleCellExperiment one can use the method
pseudobulk_lognorm <_ singIST::pseudobulk_sce(human, “celltype”, “sampleID”)

The SingleCellExperiment object should have been previously log-normalized.

- Ensure that the disease model scRNA-seq object contains standardized metadata fields. The disease model input is a Seurat or SingleCellExperiment object and must contain:

- class (experimental group membership; base vs. target)
- celltype_cluster (model clusters used to map to human cell types).
- donor (sample/donor identifier).

### 2.2 asmbPLS-DA fitting and validation

The first function the user will use is fitOptimal(), or its wrapper multiple_fitOptimal() if multiple superpathways are to be analyzed for the same human reference. The input of fitOptimal() is a superpathway_input list and its output is a fitted-model list containing the optimal model, optimal hyperparameters and validation metrics. Before calling the function, the user must define the superpathway_input list.

• First, initialize a hyperparameters list to define the hyperparameter grid and cross-validation (CV) design.
quantile_comb_table <_ as.matrix(
  RcppAlgos::permuteGeneral(
   seq(0.05, 0.95, by = 0.50), m = length(my_superpathway$celltypes), repetition = TRUE
  .)
)
my_hyperparameters <_ create_hyperparameters(quantile_comb_table =
  quantile_comb_table,
  outcome_type = “binary”,
  number_PLS = as.integer(3),
folds_CV = as.integer(1))

We have specified a LOOCV approach as folds_CV = 1, which we recommend for small sample sizes. The user must provide a grid of sparsity quantile combinations to test across cell-type blocks; the number of combinations grows exponentially with the number of modeled cell types and increases as the quantile step becomes smaller. Therefore, users should balance grid resolution with computational cost when defining quantile_comb_table.

• Second, initialize a superpathway_input list.
my_superpathway_input <_ create_superpathway_input(
superpathway_info = my_superpathway,
hyperparameters_info = my_hyperparameters,
pseudobulk_lognorm = pseudobulk_lognorm,
sample_id = c(“AD1”, “AD2”, “HC1”, “HC2″),
sample_class = c(“AD”, “AD”, “HC”, “HC”),
base_class = “HC”,
target_class = “AD”)

If the user aims to analyze only one superpathway, fitOptimal() will suffice; otherwise, if a list of superpathway_input objects is provided, call multiple_fitOptimal(). We exemplify a fitOptimal() call with parallelization (available when the CV design is LOOCV), jackknife-based variability estimation, and permutation testing for gene-importance and global model significance.

Parallelization options will vary based on OS
library(BiocParallel)
BiocParallel::register(BiocParallel::SnowParam(workers = 2, exportglobals = FALSE,
progressbar = TRUE), default = TRUE)
model <_ fitOptimal(my_superpathway_input,
parallel = TRUE,
type = “jackknife”,
nperm = 100)
# Disable parallelization
BiocParallel::register(BiocParallel::SerialParam(), default = TRUE)

Internally, fitOptimal() separates hyperparameter selection from validation to minimize optimistic model-selection bias. Cross-validation is used exclusively to select the optimal sparsity quantile combination: each candidate combination in the grid is evaluated by CV, and the combination yielding the best cross-validated classification performance is retained. The final model is then fitted on the full dataset using the selected hyperparameters. Only after this model is fitted does permutation testing assess gene-level importance and global model significance, so that the validation step does not influence hyperparameter selection. When the number of samples per class is small (fewer than three), LOOCV (folds_CV = 1) is used in place of repeated k -fold CV to maximize the training set size in each fold.

### 2.3 Recapitulation analysis

The second function the user will use is singISTrecapitulations(). This function receives a superpathway_fit_model list and mapping_organism list and returns a list containing: recapitulation metrics (superpathway, cell type) and gene contributions, and orthology mapping for each superpathway. This step requires:

a fitted model list returned by fitOptimal()/multiple_fitOptimal()
a mapping_organism list created with create_mapping_organism(), which bundles the disease model information to be used
my_mapping_organism <_ create_mapping_organism(organism = “Mus musculus”,
target_class = “ETOH”,
base_class = “OXA”, celltype_mapping =
celltype_mapping,
counts = object)

The target_class and base_class slots are values contained in celltype_cluster variable, and object refers to either a Seurat or SingleCellExperiment object. We call singISTrecapitulations() if only one superpathway is to be assessed against the mapping_organism list, otherwise call multiple_singISTrecapitulations().

recapitulations <_ singISTrecapitulations(model, my_mapping_organism)

Internally, singISTrecapitulations() computes superpathway and cell type recapitulations, as well as gene level contributions, and returns the orthology mapping used for each superpathway.

### 2.4 Visualizing results

To support interactive exploration of the high-dimensional outputs generated by singIST, we provide singIST Visualizer, a Quarto-based Shiny dashboard that consumes the results of the core analysis and renders publication-ready plots and tables. While optional, the Visualizer is designed to facilitate rapid interrogation, comparison, and sharing of results across pathways and disease models. The Visualizer is distributed as a single Quarto document with Shiny runtime and is launched after installing singIST. It accepts two standardized inputs produced by the package: (1) the fitted optimal models, obtained directly as the output of multiple_fitOptimal(), and (2) the rendered recapitulation tables, obtained by applying render_multiple_outputs() to the output of multiple_singISTrecapitulations(). Both objects are saved as.RData files and uploaded through the Visualizer interface. The Visualizer identifies each uploaded object by its internal structure rather than by variable name, so files saved under arbitrary names are recognized automatically; invalid or malformed files trigger informative error messages. A “Load Example Data” button allows users to explore the Visualizer immediately using pre-computed example results distributed with the Visualizer repository, without running the full workflow first. The following code generates both files:

# Generate the two.RData files for the singIST Visualizer
superpathways_fit <_ multiple_fitOptimal (super pathway_inputs)
recapitulations <_ multiple_sing ISTrecapitulations (disease_model, superpathways_fit)
rendered <_ render_multiple_outputs (recapitulations)
save(superpathways_fit, file = “optimal_models. RData”)
save(rendered, file = “recapitulations.RData”)

The dashboard is organized into four main panels. The Data Exploration panel allows inspection of human pseudobulk inputs and pathway composition, enabling users to assess gene-set size, block structure, and expression distributions for selected gene and cell type combinations. The Optimal Model panel summarizes model performance and feature importance, presenting class-level separation, cell type contributions, and ranked gene drivers to support interpretation of predictive signals. The Recapitulations panel displays pathway-, cell type-, and gene-level recapitulation metrics, including superpathway recapitulation scores, orthology coverage, and gene-level contributions alongside model fold changes, enabling detailed cross-species comparison. The Simulation panel is a self-contained module that does not require prior analysis outputs: it provides controlled exploration of how recapitulation scores respond to key properties of the disease model (effect-size interval, proportion of differentially expressed genes, cell-type mapping completeness, and orthology coverage) and includes a benchmark comparing singIST against a simple overlapping-differentially-expressed-gene (ODEG) approach. By integrating these complementary views, the singIST Visualizer transforms raw analysis outputs into an intuitive and reproducible user experience, enabling systematic interpretation, hypothesis generation, and automated reporting of single-cell disease model evaluations. A detailed user manual for the singIST Visualizer, including annotated screenshots, a plain-language explanation of the simulation module, and a complete end-to-end reproducible example, is provided as Supplementary Material S6.

## 3 Results

We showcase the singIST library and visualizer using the test datasets distributed with the package. These include: (1) a human scRNA-seq reference summarized as cell-type-resolved pseudobulk log-normalized expression profiles comparing healthy control and lesional atopic dermatitis skin, and (2) a disease model scRNA-seq dataset (Seurat object) annotated with donor, condition (OXA vs. ETOH), and model-specific cell type clusters.

Two representative superpathways were selected from curated MsigDB collections, and asmbPLS-DA human reference models were fitted using cross-validation and permutation-based validation with the parameters described in the vignette. The fitted reference models were then used to quantify disease model-to-human recapitulation at the superpathway, cell type, and gene levels. All outputs are stored in standardized objects and rendered for downstream visualization and interactive exploration using the singIST Visualizer.

Supplementary Figure S1 illustrates superpathway- and cell-type-level recapitulations for the oxazolone mouse model compared against the human atopic dermatitis reference. At the superpathway level, the oxazolone model shows moderate recapitulation for the Dendritic Cells in Th1/Th2 Development [BIOCARTA] superpathway (52.4%), whereas recapitulation for Cytokine-cytokine receptor interaction [KEGG] is near null (8.5%).

Inspection of cell-type-level recapitulations reveals heterogeneous contributions underlying these aggregate scores. For the BIOCARTA superpathway, recapitulation is driven by strong aligned shifts in Langerhans cells (186.3%) and dendritic cells (90.9%), partially counterbalanced by an opposite contribution from T cells (−84.1%). In contrast, the KEGG superpathway shows strong positive recapitulation in T cells and dendritic cells (365.1% and 133.7%, respectively), offset by negative recapitulations in keratinocytes (−261.9%) and Langerhans cells (−101.3%). These results illustrate how superpathway-level agreement can arise from opposing cell-type-specific effects.

Recapitulation scores are signed and unbounded, so values below 0% or above 100% carry specific meaning rather than indicating error. A value near 100% indicates that the disease model reproduces the human effect at comparable magnitude and direction; values above 100% indicate that the disease model overshoots the human effect (larger fold change in the same direction); and values below 0% indicate that the disease model shifts in the opposite direction to the human reference. Values near 0% reflect absence of recapitulation. This interpretation is supported by the simulation-based validation described below, in which aligned and opposed disease-model effects yield recapitulation scores centered near 100% and −100%, respectively.

### 3.1 Simulation-based validation

To complement the applied case study and to probe the behavior of singIST under controlled conditions, we implemented a self-contained simulation module distributed with the singIST Visualizer. The module generates synthetic human pseudobulk reference data and disease-model single-cell data with tunable properties, constructs the corresponding singIST inputs, and computes recapitulation scores without requiring external databases, so that validation can be run entirely locally.

Five property-exploration cases examine how recapitulation responds to key disease-model properties: (i) the interval of disease-model log2 fold changes relative to a fixed human effect; (ii) the proportion of genes declared differentially expressed; (iii) the number of cell types retained in the cell-type mapping (cell-type dropout); (iv) the percentage of genes retained after orthology mapping; and (v) the alignment or opposition of disease-model and human effect directions across a range of fold-change magnitudes. Across these cases, recapitulation scores are approximately 100% when disease-model and human effects are aligned and of comparable magnitude, approach −100% when the effects are opposed, decrease toward 0% as cell types are dropped from the mapping or orthology coverage is reduced, and scale with the proportion of differentially expressed genes.

A benchmark case compares singIST against a simple overlapping-differentially-expressed-gene (ODEG) approach, the *de facto* standard for single-cell model-to-human comparison (Kim et al., 2019; Li et al., 2023; Ali et al., 2024), that intersects genes declared significant in both the human reference and the disease model. Three scenarios are considered: agreement, direction disagreement, and magnitude disagreement between the two data sources. singIST captures both the direction and the relative magnitude of effects through its signed recapitulation scores, whereas the ODEG approach reports only the count of shared significant genes and is insensitive to whether the shared genes shift in the same or opposite directions, or to differences in effect size, limitations that have been documented previously (Lawhorn et al., 2018). The simulation module and benchmark are accessible through the Simulation panel of the singIST Visualizer and as standalone functions distributed with the singIST Visualizer repository.

A fully reproducible vignette describing this analysis is provided in Supplementary Material S2.

## 4 Conclusion

*singIST* provides a practical software solution for systematic and interpretable comparison of disease model single-cell transcriptomic data against human reference datasets. By incorporating a previously described methodological framework into a standardized R/Bioconductor package, singIST enables reproducible assessment of biological recapitulation across pathways, cell types, and genes, while reducing the technical burden associated with implementing complex comparative analyses. The simulation module included with the singIST Visualizer further provides a controlled, parameter-tunable environment for validating method behavior and benchmarking against simpler overlap-based approaches.

In addition, the companion singIST Visualizer facilitates interactive exploration and reporting of high-dimensional results, supporting interpretation and communication without requiring custom static visualization workflows.

Together, singIST and its Visualizer support informed evaluation of preclinical disease models in applied translational research settings, bridging the gap between methodological sophistication and practical usability. By promoting standardization, reproducibility, and accessibility, singIST enables broader adoption of comparative single-cell transcriptomic analyses for model assessment and hypothesis generation.

## 5 Data privacy and security

The singIST Visualizer can be run either locally or through a hosted Shiny instance. When run locally, all uploaded files are loaded directly into the user’s R session and no data are transmitted to or stored on any external server. This is the recommended mode for sensitive or unpublished datasets.

For the hosted Shiny instance, the following data handling practices apply. Uploaded.RData files are temporarily loaded into the server-side R session to generate visualizations. These files are retained only for the duration of the active session and are automatically deleted when the session ends or times out; no data are persisted, backed up, or retained after session termination. The server does not log or store the contents of uploaded files. Communication between the client browser and the server uses HTTPS encryption. Server access is restricted to authorized maintainers for administrative purposes only.

A privacy notice summarizing these practices is displayed on the Visualizer upload page before users submit a file. Users are explicitly advised to run the Visualizer locally if their data are sensitive or subject to institutional or regulatory data-protection requirements. The singIST package itself does not transmit any data externally except during the orthology mapping step, which queries the public Ensembl BioMart API; no user data are sent in this query, only gene identifiers for orthology lookup.

## Data Availability

The original contributions presented in the study are included in the article/Supplementary Material, further inquiries can be directed to the corresponding author.
