# Supplementary material for: singIST: an R/bioconductor library and quarto dashboard for automated single-cell comparative transcriptomics analysis of disease models and humans
Source: Front Genet. 2026 Sep 25;17:1952333. doi: 10.3389/fgene.2026.1952333 (PMC13649217; doi:10.3389/fgene.2026.1952333)
Supplement: Supplementary file 1 [file DataSheet1.pdf]

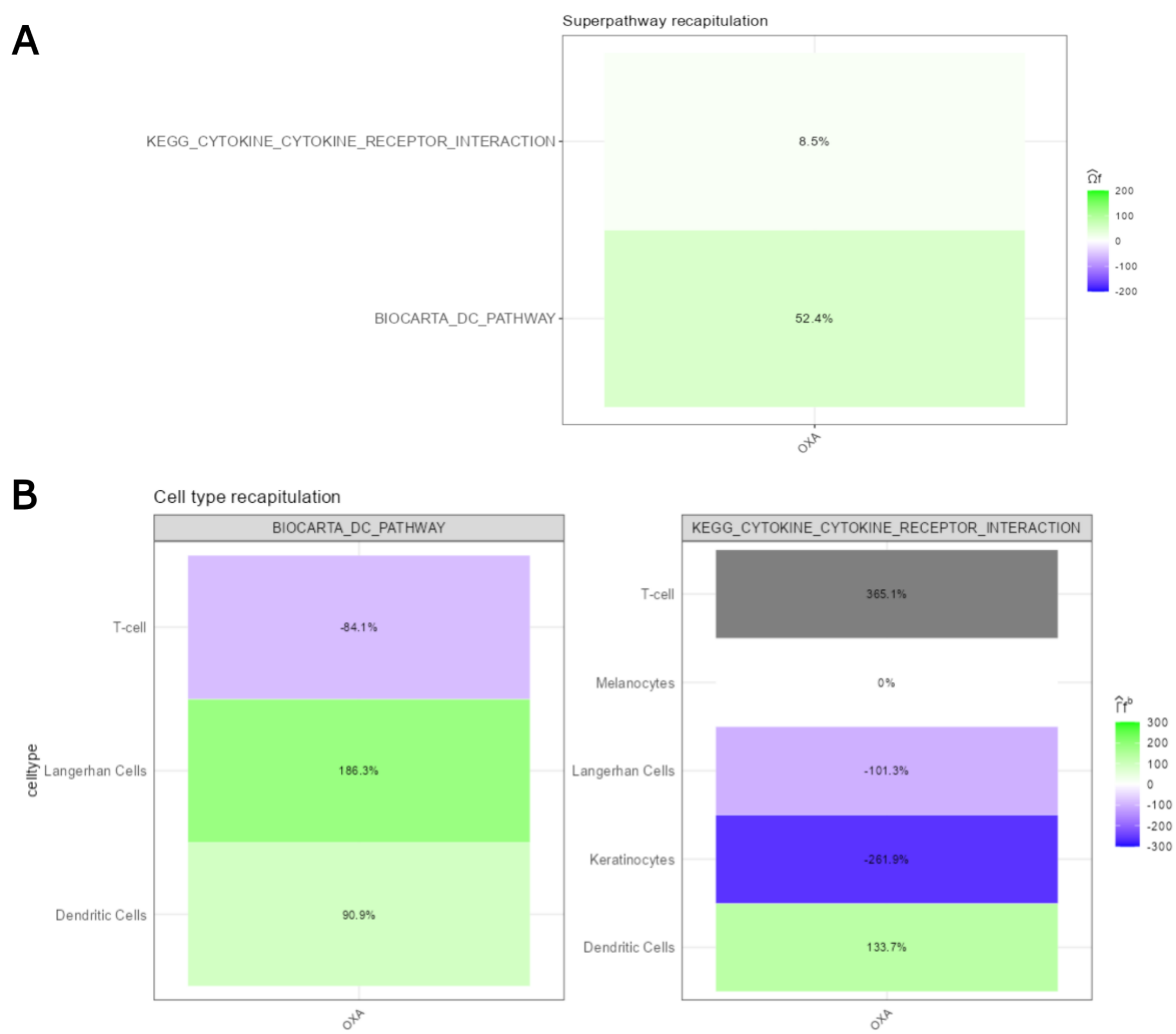

Figure 1: **Oxazolone mouse model recapitulations against Atopic Dermatitis human reference. A) Superpathway recapitulation ($\widehat{\Omega}f$).** Signed fractional recapitulation at the superpathway level for two superpathways in the oxazolone (OXA) model. Values quantify how closely disease model induced changes align with the direction and magnitude of the human reference signal; negative values indicate opposite direction shifts and values greater than 100% indicate larger magnitude shift. **B) Cell type recapitulation ($\widehat{\Gamma}f$)** Cell type signed fractional recapitulation for the same two superpathways. The heatmaps highlight which cell types drive superpathway level agreement or disagreement, and reveal opposing recapitulations that can partially cancel at the superpathway level. Figures generated with singIST Visualizer.
