# Supplementary material for: singIST: an R/bioconductor library and quarto dashboard for automated single-cell comparative transcriptomics analysis of disease models and humans
Source: Front Genet. 2026 Sep 25;17:1952333. doi: 10.3389/fgene.2026.1952333 (PMC13649217; doi:10.3389/fgene.2026.1952333)
Supplement: Supplementary file 2 [file DataSheet2.pdf]

singIST Visualizer User Manual

1. Overview

The singIST Visualizer is a Quarto-based Shiny application for interactive exploration of singIST analysis results. It provides four panels:

1. Data Exploration: inspect human pseudobulk inputs and pathway composition.
2. Optimal Model: summarize model performance and feature importance.
3. Recapitulations: display pathway, cell type, and gene-level recapitulation.
4. Simulation: self-contained parameter exploration and benchmarking (no upload required).

2. Launching the Visualizer

Local launch (recommended for sensitive data)

```
# Install singIST and Quarto
# BiocManager::install("singIST")
# install.packages("quarto")

# Clone the visualizer repository
# git clone https://github.com/DataScienceRD-Almirall/singIST-visualizer

# Launch from R
quarto::quarto_run("path/to/singIST_visualizer.qmd")
```

Alternatively, open `singIST_visualizer.qmd` in RStudio and click Render.

Hosted version

A hosted version is available at: https://amoruno.shinyapps.io/singIST_visualizer/ (https://amoruno.shinyapps.io/singIST_visualizer/)

> Note: for sensitive or unpublished datasets, always run the Visualizer locally. See the Data Privacy Notice on the upload page and the Data Privacy and Security section of the manuscript.

3. Uploading Data

The Visualizer requires two `.RData` files:

File	Content	Generated by
Optimal Models (.RData)	Named list of fitted models	`multiple_fitOptimal()`
Recapitulations (.RData)	Rendered plot-ready tables	`render_multiple_outputs()`

How to upload

1. Navigate to the Uploading sidebar on the left side of the application.
2. Click “Upload Optimal Models (.RData)” and select your optimal models file.
3. Click “Upload Recapitulations (.RData)” and select your recapitulations file.
4. A green notification confirms successful upload. A red notification indicates an error (see Section 6 for troubleshooting).

Object identification

The Visualizer identifies uploaded objects by their internal structure, not by variable name. This means you can save your results under any variable name:

```
# These are all valid -- the Visualizer will find the correct object
save(my_models, file = "optimal_models.RData")
save(fit_results, file = "optimal_models.RData")
save(superpathways_fit, file = "optimal_models.RData")
```

Load Example Data

If you want to explore the Visualizer without running the full singIST workflow:

1. Click the “Load Example Data” button on the upload page.
2. Pre-computed example results are loaded from the Visualizer’s local `extdata/` directory (`extdata/optimal_models.RData` and `extdata/recapitulations.RData`).
3. If the example files are not found, generate them first by running the script distributed with the Visualizer repository:

```
# From the singIST-visualizer repository root
# Requires an active Ensembl connection for orthology mapping
source("generate_test_data_3pathways.R")
```

`generate_test_data_3pathways.R` loads the example data distributed with the singIST package, runs three pathways that all share the same set of cell types (Dendritic Cells, Langerhan Cells, T-cell) against the oxazolone mouse model, and saves both `.RData` files to `extdata/` . An earlier single-pathway helper, `generate_visualizer_example_data.R` , is also available. After generating the files, click “Load Example Data” to load them into the Visualizer.

4. Panel Descriptions

4.1 Data Exploration Panel

Purpose: inspect the human pseudobulk reference data and pathway composition.

Controls:

- Choose Superpathway: dropdown to select a superpathway.
- Choose Cell Type: dropdown to filter by cell type.
- Choose Gene: dropdown to select a specific gene.

Visualizations:

- Gene-set size plot: bar chart showing the number of genes per cell type block for the selected superpathway.

- PCA and boxplot: PCA scatter plot showing sample separation colored by class, and expression boxplots for the selected gene and cell type.

4.2 Optimal Model Panel

Purpose: summarize model performance and feature importance.

Controls:

- Choose Superpathway: dropdown to select a superpathway.

Visualizations:

- Class separation plot: projection of samples onto the first two PLS components, colored by class.
- Cell type contributions: bar chart showing the relative contribution of each cell type block to the model.
- Gene drivers: ranked bar chart of the most important genes driving class separation.

4.3 Recapitulations Panel

Purpose: display recapitulation metrics at pathway, cell type, and gene level.

Controls:

- Choose Superpathway: dropdown to select a superpathway.
- Choose Target Organism: dropdown to select the disease model organism.

Visualizations:

- Superpathway recapitulation: bar chart of recapitulation scores across superpathways and disease models.
- Cell type recapitulation: heatmap of cell-type-level recapitulation scores.
- Gene-level contributions: scatter plot of gene-level fold changes (disease model vs. human reference), colored by orthology status.

4.4 Simulation Panel

Purpose: explore how recapitulation scores respond to disease model properties and benchmark singIST against a simple overlapping-DEG approach. This panel does not require uploaded data.

Controls:

- Property Exploration: dropdown to select one of five simulation cases:
  1. Effect-size interval.
  2. Proportion of differentially expressed genes.
  3. Cell-type dropout.
  4. Orthology coverage.
  5. Alignment vs. opposition of effects.
- ODEG Benchmark: dropdown to select one of three benchmark scenarios:
  1. Agreement.
  2. Direction disagreement.
  3. Magnitude disagreement.

Visualizations:

- Property exploration: line plots showing recapitulation scores as a function of the varied parameter.
- ODEG benchmark: Venn diagrams of overlapping DEGs and a heatmap of singIST recapitulation scores.

Programmatic access: the simulation functions are also available outside the Visualizer via `simulation_helpers.R` (sourced automatically by the `.qmd`). A standalone example script, `simulation_example.R`, demonstrates all five property cases and the three ODEG benchmark scenarios with publication-ready plots. Run it from the repository root:

```
source("simulation_helpers.R")
# then run simulation_example.R, or call run_simulation_case() / run_odeg_benchmark() directl
y
```

A plain-language explanation of what each simulation case and benchmark scenario shows, and why it matters, is provided in Section 10.

5. Exporting Figures and Tables

All plots in the Visualizer are rendered using ggplot2 and ComplexHeatmap. To export:

- Plots: use the Shiny plot export menu (hover over the plot and click the camera icon) to download as PNG or PDF.
- Tables: where tables are displayed, use the DataTable export buttons to download as CSV or Excel.

6. Troubleshooting Upload Errors

Error message	Cause	Solution
"Uploaded object is not a list"	File contains a non-list object	Ensure you saved the output of `multiple_fitOptimal()` or `render_multiple_outputs()`
"Missing required elements"	Object is missing expected fields	Re-run `render_multiple_outputs()` on your recapitulations output
"No object matches the expected singIST structure"	Multiple objects in file, none valid	Save only the singIST output object in the .RData file
"No objects found in the .RData file"	Empty or corrupted file	Re-save the file using `save()`
"Example .RData files not found"	Example data not generated	Run `generate_test_data_3pathways.R`
"Error loading file: …"	File is not a valid .RData format	Ensure the file was created with `save()` in R

7. Data Privacy

- Local mode: no data leaves your machine. All processing happens in your R session.
- Hosted mode: uploaded files are temporarily processed server-side and automatically deleted at session end. No data is retained afterward.
- Recommendation: for sensitive or unpublished datasets, run the Visualizer locally.

See the Data Privacy and Security section of the manuscript for full details.

8. End-to-End Reproducible Example

This section demonstrates the complete workflow from loading the example datasets distributed with the singIST package to generating the two `.RData` files required by the Visualizer and launching the application. The example uses three pathways that all share the same set of cell types, so that the human pseudobulk reference is identical across pathways and the three superpathways can be compared on a common footing. The code mirrors the singIST package vignette and can be run directly in R; it is also available as the ready-to-run script `generate_test_data_3pathways.R` in the Visualizer repository.

Step 1: Load packages and define the common cell types

```
library(singIST)
library(msigdb)
library(RcppAlgos)
library(BiocParallel)
set.seed(42)

# Identical cell types across all three pathways
common_celltypes <- c("Dendritic Cells", "Langerhan Cells", "T-cell")
```

Step 2: Prepare a single merged human pseudobulk reference

The singIST package ships two pseudobulk matrices, `DC_counts.rda` and `cytokine_counts.rda`. We keep only the common cell types and merge them by the union of genes, producing one reference matrix (27 rows = 3 cell types x 9 samples; 270 genes) used for every pathway.

```
extdata_dir <- system.file("extdata", package = "singIST")
load(file.path(extdata_dir, "DC_counts.rda"))        # DC_counts       (54 x 17)
load(file.path(extdata_dir, "cytokine_counts.rda"))  # cytokine_counts (54 x 263)

keep_rows <- function(m)
    m[sub("_.*$", "", rownames(m)) %in% common_celltypes, , drop = FALSE]

dc_sub <- keep_rows(DC_counts)
cy_sub <- keep_rows(cytokine_counts)

all_genes <- union(colnames(dc_sub), colnames(cy_sub))
align <- function(m, g) {
    out <- matrix(0, nrow(m), length(g),
                  dimnames = list(rownames(m), g))
    out[, colnames(m)] <- m
    out
}
merged <- rbind(align(dc_sub, all_genes), align(cy_sub, all_genes))
merged <- log2(merged + 1)   # pseudobulk log-normalization
available_genes <- colnames(merged)
```

Step 3: Build the three superpathway inputs

Each pathway is resolved from a list of candidate (standard_name, dbsource) pairs, tried in order, so the script is robust to MSigDB version differences. The gene set is assigned to every cell type with `setGeneSetsCelltype()`.

```r
library(msigdb)
gse <- msigdb::getMsigdb(org = "hs", id = "SYM",
                         collection = "c2", subcollection = "CP")

available_names <- function(db) {
    gse_sub <- msigdb::subsetCollection(gse, collection = "c2",
                                        subcollection = db)
    names(gse_sub)
}

# Helper: resolve a pathway from candidate (standard_name, dbsource) pairs,
# tried in order. Returns list(pw, genes) for the first candidate whose gene
# set can be pulled. Robust to MSigDB version differences (e.g. KEGG_LEGACY
# vs KEGG_MEDICUS): some KEGG pathway names move between versions.
resolve_pathway <- function(candidates) {
    for (cand in candidates) {
        pw <- create_pathway(standard_name = cand[1], dbsource = cand[2],
                             collection = "c2", subcollection = "CP")
        genes <- tryCatch(pullGeneSet(pw, gse = gse), error = function(e) NULL)
        if (!is.null(genes) && length(genes) > 0) {
            message("  Using pathway: ", cand[1], " [", cand[2], "] (",
                    length(genes), " genes)")
            return(list(pw = pw, genes = genes))
        }
    }
    stop("None of the candidate pathways were found in this MSigDB version.\n",
         "Tried: ", paste(sapply(candidates, `[`, 1), collapse = ", "),
         "\nAvailable names for the last subcollection (",
         candidates[[length(candidates)]][2], "):\n  ",
         paste(head(available_names(candidates[[length(candidates)]][2]), 50),
               collapse = "\n  "),
         "\nPick one and add it to the candidate list.")
}

# Each pathway is a list of candidate (standard_name, dbsource) pairs, tried
# in order. The first candidate that exists in the loaded MSigDB version is
# used. Slot 3 uses REACTOME (always present in base MSigDB) as the primary
# candidate, with KEGG and BIOCARTA fallbacks.
pathway_candidates <- list(
    list(c("BIOCARTA_DC_PATHWAY", "BIOCARTA")),
    list(c("KEGG_CYTOKINE_CYTOKINE_RECEPTOR_INTERACTION", "KEGG")),
    list(c("REACTOME_CYTOKINE_SIGNALING_IN_IMMUNE_SYSTEM", "REACTOME"),
         c("KEGG_TH1_AND_TH2_CELL_DIFFERENTIATION",        "KEGG"),
         c("BIOCARTA_TH1TH2_PATHWAY",                      "BIOCARTA")))

sample_id    <- c("AD1", "AD13", "AD2", "AD3", "AD4", "HC1-2", "HC3", "HC4", "HC5")
sample_class <- c(rep("AD", 5), rep("HC", 4))

# sparsity quantile grid for 3 cell-type blocks (27 combinations)
quantile_grid <- as.matrix(
    RcppAlgos::permuteGeneral(seq(0.1, 0.95, by = 0.3),
                              m = length(common_celltypes), repetition = TRUE))

superpathway_inputs <- list()
for (cands in pathway_candidates) {
```

```r
    rp <- resolve_pathway(cands)
    pw <- rp$pw
    genes <- rp$genes

    sp <- create_superpathway(pathway_info = pw,
                              celltypes = common_celltypes,
                              gene_sets_celltype = list())
    # assign the gene set to every cell type via the official API
    # (setGeneSetsCelltype with value=NULL pulls from MSigDB internally)
    sp <- setGeneSetsCelltype(sp, gse = gse)
    # keep only genes present in the merged pseudobulk
    sp$gene_sets_celltype <- lapply(sp$gene_sets_celltype,
                                    function(gs) intersect(gs, available_genes))

    hp <- create_hyperparameters(quantile_comb_table = quantile_grid,
                                 outcome_type = "binary",
                                 number_PLS = 2L, folds_CV = 1L, repetition_CV = 1L)

    superpathway_inputs[[pw$standard_name]] <- create_superpathway_input(
        superpathway_info    = sp,
        hyperparameters_info = hp,
        pseudobulk_lognorm   = merged,
        sample_id            = sample_id,
        sample_class         = sample_class,
        base_class           = "HC",
        target_class         = "AD")
}
```

Step 4: Prepare the disease model mapping organism (OXA)

```r
load(file.path(extdata_dir, "OXA.rda"))  # OXA (Seurat object)
colnames(slot(OXA, "meta.data"))[c(5, 11, 1)] <- c("class",
                                                   "celltype_cluster",
                                                   "donor")
oxa_org <- create_mapping_organism(
    organism = "Mus musculus",
    target_class = "OXA", base_class = "ETOH",
    celltype_mapping = list(
        "Dendritic Cells" = c("cDC2", "cDC1", "migratory DCs"),
        "Langerhan Cells" = c("LC"),
        "T-cell"          = c("DETC", "T")),
    counts = OXA)
```

Step 5: Fit optimal models, compute recapitulations, and render

```
BiocParallel::register(
    BiocParallel::SnowParam(workers = 2, exportglobals = FALSE,
                            progressbar = FALSE), default = TRUE)

superpathways_fit <- multiple_fitOptimal(
    superpathway_inputs,
    parallel     = rep(TRUE, length(superpathway_inputs)),
    npermut      = 100,
    type         = rep("jackknife", length(superpathway_inputs)),
    nsubsampling = NULL)

BiocParallel::register(BiocParallel::SerialParam(), default = TRUE)

# Requires an active Ensembl connection for mouse-to-human orthology mapping
recapitulations <- multiple_singISTrecapitulations(oxa_org, superpathways_fit)
rendered <- render_multiple_outputs(objects = list(recapitulations))
```

Step 6: Save the .RData files for the Visualizer

```
out_dir <- "extdata"
if (!dir.exists(out_dir)) dir.create(out_dir, recursive = TRUE)
save(superpathways_fit, file = file.path(out_dir, "optimal_models.RData"))
save(rendered,          file = file.path(out_dir, "recapitulations.RData"))
```

Step 7: Launch the Visualizer and load data

```
# Option A: Launch locally
quarto::quarto_run("singIST_visualizer.qmd")

# Option B: Use the hosted version
# https://amoruno.shinyapps.io/singIST_visualizer/
```

Then either upload the two `.RData` files via the upload controls, or click the “Load Example Data” button to use the pre-computed example results (generate them first with `generate_test_data_3pathways.R` if needed).

Quick start: example data only

If you do not need to run the full workflow and just want to explore the Visualizer with pre-computed results:

```
# From the singIST-visualizer repository root
source("generate_test_data_3pathways.R")      # creates extdata/*.RData
quarto::quarto_run("singIST_visualizer.qmd")  # then click "Load Example Data"
```

singIST Visualizer User Manual

1. Overview

The singIST Visualizer is a Quarto-based Shiny application for interactive exploration of singIST analysis results. It provides four panels:

1. Data Exploration: inspect human pseudobulk inputs and pathway composition.
2. Optimal Model: summarize model performance and feature importance.
3. Recapitulations: display pathway, cell type, and gene-level recapitulation.
4. Simulation: self-contained parameter exploration and benchmarking (no upload required).

2. Launching the Visualizer

Local launch (recommended for sensitive data)

```
# Install singIST and Quarto
# BiocManager::install("singIST")
# install.packages("quarto")

# Clone the visualizer repository
# git clone https://github.com/DataScienceRD-Almirall/singIST-visualizer

# Launch from R
quarto::quarto_run("path/to/singIST_visualizer.qmd")
```

Alternatively, open `singIST_visualizer.qmd` in RStudio and click Render.

Hosted version

A hosted version is available at: https://amoruno.shinyapps.io/singIST_visualizer/ (https://amoruno.shinyapps.io/singIST_visualizer/)

> Note: for sensitive or unpublished datasets, always run the Visualizer locally. See the Data Privacy Notice on the upload page and the Data Privacy and Security section of the manuscript.

3. Uploading Data

The Visualizer requires two `.RData` files:

File	Content	Generated by
Optimal Models (.RData)	Named list of fitted models	`multiple_fitOptimal()`
Recapitulations (.RData)	Rendered plot-ready tables	`render_multiple_outputs()`

How to upload

1. Navigate to the Uploading sidebar on the left side of the application.
2. Click "Upload Optimal Models (.RData)" and select your optimal models file.
3. Click "Upload Recapitulations (.RData)" and select your recapitulations file.

4. A green notification confirms successful upload. A red notification indicates an error (see Section 6 for troubleshooting).

Object identification

The Visualizer identifies uploaded objects by their internal structure, not by variable name. This means you can save your results under any variable name:

```
# These are all valid -- the Visualizer will find the correct object
save(my_models, file = "optimal_models.RData")
save(fit_results, file = "optimal_models.RData")
save(superpathways_fit, file = "optimal_models.RData")
```

Load Example Data

If you want to explore the Visualizer without running the full singIST workflow:

1. Click the "Load Example Data" button on the upload page.
2. Pre-computed example results are loaded from the Visualizer's local `extdata/` directory (`extdata/optimal_models.RData` and `extdata/recapitulations.RData`).
3. If the example files are not found, generate them first by running the script distributed with the Visualizer repository:

```
# From the singIST-visualizer repository root
# Requires an active Ensembl connection for orthology mapping
source("generate_test_data_3pathways.R")
```

`generate_test_data_3pathways.R` loads the example data distributed with the singIST package, runs three pathways that all share the same set of cell types (Dendritic Cells, Langerhan Cells, T-cell) against the oxazolone mouse model, and saves both `.RData` files to `extdata/` . An earlier single-pathway helper, `generate_visualizer_example_data.R` , is also available. After generating the files, click "Load Example Data" to load them into the Visualizer.

4. Panel Descriptions

4.1 Data Exploration Panel

Purpose: inspect the human pseudobulk reference data and pathway composition.

Controls:

- Choose Superpathway: dropdown to select a superpathway.
- Choose Cell Type: dropdown to filter by cell type.
- Choose Gene: dropdown to select a specific gene.

Visualizations:

- Gene-set size plot: bar chart showing the number of genes per cell type block for the selected superpathway.
- PCA and boxplot: PCA scatter plot showing sample separation colored by class, and expression boxplots for the selected gene and cell type.

4.2 Optimal Model Panel

Purpose: summarize model performance and feature importance.

Controls:

- Choose Superpathway: dropdown to select a superpathway.

Visualizations:

- Class separation plot: projection of samples onto the first two PLS components, colored by class.
- Cell type contributions: bar chart showing the relative contribution of each cell type block to the model.
- Gene drivers: ranked bar chart of the most important genes driving class separation.

4.3 Recapitulations Panel

Purpose: display recapitulation metrics at pathway, cell type, and gene level.

Controls:

- Choose Superpathway: dropdown to select a superpathway.
- Choose Target Organism: dropdown to select the disease model organism.

Visualizations:

- Superpathway recapitulation: bar chart of recapitulation scores across superpathways and disease models.
- Cell type recapitulation: heatmap of cell-type-level recapitulation scores.
- Gene-level contributions: scatter plot of gene-level fold changes (disease model vs. human reference), colored by orthology status.

4.4 Simulation Panel

Purpose: explore how recapitulation scores respond to disease-model properties and benchmark singIST against a simple overlapping-DEG approach. This panel does not require uploaded data.

Controls:

- Property Exploration: dropdown to select one of five simulation cases:
    1. Effect-size interval.
    2. Proportion of differentially expressed genes.
    3. Cell-type dropout.
    4. Orthology coverage.
    5. Alignment vs. opposition of effects.
- ODEG Benchmark: dropdown to select one of three benchmark scenarios:
    1. Agreement.
    2. Direction disagreement.
    3. Magnitude disagreement.

Visualizations:

- Property exploration: line plots showing recapitulation scores as a function of the varied parameter.
- ODEG benchmark: Venn diagrams of overlapping DEGs and a heatmap of singIST recapitulation scores.

Programmatic access: the simulation functions are also available outside the Visualizer via `simulation_helpers.R` (sourced automatically by the `.qmd`). A standalone example script, `simulation_example.R`, demonstrates all five property cases and the three ODEG benchmark scenarios with publication-ready plots. Run it from the repository root:

```
source("simulation_helpers.R")
# then run simulation_example.R, or call run_simulation_case() / run_odeg_benchmark() directly
```

A plain-language explanation of what each simulation case and benchmark scenario shows, and why it matters, is provided in Section 10.

5. Exporting Figures and Tables

Every results panel of the Visualizer provides a standardized export row directly above the output, with the same controls in all tabs:

- **PNG**, **PDF** and **SVG** buttons export the figure. PNG exports are rendered at 300 dpi, PDF (cairo) and SVG (svglite) exports are vector graphics, suitable for publication. The canvas size of each exported figure is computed automatically from its content.
- **CSV** button, which exports the exact data underlying the panel.

The two interactive lookup tables (GIP table and gene contribution table) instead use the DataTable **Copy/CSV/Excel** buttons at the top-left of the table. These buttons always export the **complete table** (all rows, not just the visible page), with file names that include the singIST version and the download timestamp (singIST_v<version>_GIP_table_<YYYYMMDD-HHMMSS>.* , singIST_v<version>_gene_contribution_table_<YYYYMMDD-HHMMSS>.*).

The export area is available for all 14 results panels:

Tab	Panel	Figure	Table (CSV)
Data Exploration	Gene set size distribution	PNG/PDF/SVG	Yes
Data Exploration	PCA of pseudobulk block matrix	PNG/PDF/SVG	Yes
Data Exploration	PCA of selected cell type block	PNG/PDF/SVG	Yes
Data Exploration	Gene expression boxplot (t-test)	PNG/PDF/SVG	Yes
Optimal Model	Superpathway scores	PNG/PDF/SVG	Yes
Optimal Model	Radar plot	PNG/PDF/SVG	Yes
Optimal Model	Jitter plot	PNG/PDF/SVG	Yes
Optimal Model	GIP lookup table	–	DataTable Copy/CSV/Excel buttons (full table)
Recapitulations	Superpathway recapitulation	PNG/PDF/SVG	Yes
Recapitulations	Orthology coverage	PNG/PDF/SVG	Yes
Recapitulations	Cell type recapitulation	PNG/PDF/SVG	Yes
Recapitulations	Gene contribution heatmap	PNG/PDF/SVG	Yes

Tab	Panel	Figure	Table (CSV)
Recapitulations	Gene contribution lookup table	–	DataTable Copy/CSV/Excel buttons (full table)
Simulation	Simulation results plot	PNG/PDF/SVG	Yes
Simulation	ODEG Venn diagram	PNG/PDF/SVG	Yes
Simulation	ODEG heatmap	PNG/PDF/SVG	Yes

Exported file names

All exported files follow a consistent, informative naming scheme:

```
singIST_v<package-version>_<analysis>_<context>_<YYYYMMDD-HHMMSS>.<ext>
```

where <analysis> identifies the panel (e.g. `superpathway_recap`, `celltype_recap`, `gene_contribution`, `pca_and_boxplot`), <context> identifies the pathway, cell type, or simulation case shown in the panel when applicable, and the timestamp records when the file was exported. For example:

```
singIST_v1.2.1_superpathway_recap_KEGG_CYTOKINE_CYTOKINE_RECEPTOR_INTERACTION_20260820-154530.png
singIST_v1.2.1_gene_contribution_Dendritic_Cells_20260820-154612.csv
```

This makes every exported file traceable to the singIST version, the analysis, the biological context, and the export time.

6. Troubleshooting Upload Errors

Error message	Cause	Solution
"Uploaded object is not a list"	File contains a non-list object	Ensure you saved the output of `multiple_fitOptimal()` or `render_multiple_outputs()`
"Missing required elements"	Object is missing expected fields	Re-run `render_multiple_outputs()` on your recapitulations output
"No object matches the expected singIST structure"	Multiple objects in file, none valid	Save only the singIST output object in the .RData file
"No objects found in the .RData file"	Empty or corrupted file	Re-save the file using `save()`
"Example .RData files not found"	Example data not generated	Run `generate_test_data_3pathways.R`
"Error loading file: …"	File is not a valid .RData format	Ensure the file was created with `save()` in R

7. Data Privacy

- Local mode: no data leaves your machine. All processing happens in your R session.

- Hosted mode: uploaded files are temporarily processed server-side and automatically deleted at session end. No data is retained afterward.
- Recommendation: for sensitive or unpublished datasets, run the Visualizer locally.

See the Data Privacy and Security section of the manuscript for full details.

8. End-to-End Reproducible Example

This section demonstrates the complete workflow from loading the example datasets distributed with the singIST package to generating the two `.RData` files required by the Visualizer and launching the application. The example uses three pathways that all share the same set of cell types, so that the human pseudobulk reference is identical across pathways and the three superpathways can be compared on a common footing. The code mirrors the singIST package vignette and can be run directly in R; it is also available as the ready-to-run script `generate_test_data_3pathways.R` in the Visualizer repository.

Step 1: Load packages and define the common cell types

```
library(singIST)
library(msigdb)
library(RcppAlgos)
library(BiocParallel)
set.seed(42)

# Identical cell types across all three pathways
common_celltypes <- c("Dendritic Cells", "Langerhan Cells", "T-cell")
```

Step 2: Prepare a single merged human pseudobulk reference

The singIST package ships two pseudobulk matrices, `DC_counts.rda` and `cytokine_counts.rda`. We keep only the common cell types and merge them by the union of genes, producing one reference matrix (27 rows = 3 cell types x 9 samples; 270 genes) used for every pathway.

```
extdata_dir <- system.file("extdata", package = "singIST")
load(file.path(extdata_dir, "DC_counts.rda"))        # DC_counts       (54 x 17)
load(file.path(extdata_dir, "cytokine_counts.rda"))  # cytokine_counts (54 x 263)

keep_rows <- function(m)
    m[sub("_.*$", "", rownames(m)) %in% common_celltypes, , drop = FALSE]

dc_sub <- keep_rows(DC_counts)
cy_sub <- keep_rows(cytokine_counts)

all_genes <- union(colnames(dc_sub), colnames(cy_sub))
align <- function(m, g) {
    out <- matrix(0, nrow(m), length(g),
                  dimnames = list(rownames(m), g))
    out[, colnames(m)] <- m
    out
}
merged <- rbind(align(dc_sub, all_genes), align(cy_sub, all_genes))
merged <- log2(merged + 1)   # pseudobulk log-normalization
available_genes <- colnames(merged)
```

Step 3: Build the three superpathway inputs

Each pathway is resolved from a list of candidate (standard_name, dbsource) pairs, tried in order, so the script is robust to MSigDB version differences. The gene set is assigned to every cell type with `setGeneSetsCelltype()`.

```r
library(msigdb)
gse <- msigdb::getMsigdb(org = "hs", id = "SYM",
                         collection = "c2", subcollection = "CP")

available_names <- function(db) {
    gse_sub <- msigdb::subsetCollection(gse, collection = "c2",
                                        subcollection = db)
    names(gse_sub)
}

# Helper: resolve a pathway from candidate (standard_name, dbsource) pairs,
# tried in order. Returns list(pw, genes) for the first candidate whose gene
# set can be pulled. Robust to MSigDB version differences (e.g. KEGG_LEGACY
# vs KEGG_MEDICUS): some KEGG pathway names move between versions.
resolve_pathway <- function(candidates) {
    for (cand in candidates) {
        pw <- create_pathway(standard_name = cand[1], dbsource = cand[2],
                             collection = "c2", subcollection = "CP")
        genes <- tryCatch(pullGeneSet(pw, gse = gse), error = function(e) NULL)
        if (!is.null(genes) && length(genes) > 0) {
            message("  Using pathway: ", cand[1], " [", cand[2], "] (",
                    length(genes), " genes)")
            return(list(pw = pw, genes = genes))
        }
    }
    stop("None of the candidate pathways were found in this MSigDB version.\n",
         "Tried: ", paste(sapply(candidates, `[`, 1), collapse = ", "),
         "\nAvailable names for the last subcollection (",
         candidates[[length(candidates)]][2], "):\n  ",
         paste(head(available_names(candidates[[length(candidates)]][2]), 50),
               collapse = "\n  "),
         "\nPick one and add it to the candidate list.")
}

# Each pathway is a list of candidate (standard_name, dbsource) pairs, tried
# in order. The first candidate that exists in the loaded MSigDB version is
# used. Slot 3 uses REACTOME (always present in base MSigDB) as the primary
# candidate, with KEGG and BIOCARTA fallbacks.
pathway_candidates <- list(
    list(c("BIOCARTA_DC_PATHWAY", "BIOCARTA")),
    list(c("KEGG_CYTOKINE_CYTOKINE_RECEPTOR_INTERACTION", "KEGG")),
    list(c("REACTOME_CYTOKINE_SIGNALING_IN_IMMUNE_SYSTEM", "REACTOME"),
         c("KEGG_TH1_AND_TH2_CELL_DIFFERENTIATION",        "KEGG"),
         c("BIOCARTA_TH1TH2_PATHWAY",                      "BIOCARTA")))

sample_id    <- c("AD1", "AD13", "AD2", "AD3", "AD4", "HC1-2", "HC3", "HC4", "HC5")
sample_class <- c(rep("AD", 5), rep("HC", 4))

# sparsity quantile grid for 3 cell-type blocks (27 combinations)
quantile_grid <- as.matrix(
    RcppAlgos::permuteGeneral(seq(0.1, 0.95, by = 0.3),
                              m = length(common_celltypes), repetition = TRUE))

superpathway_inputs <- list()
for (cands in pathway_candidates) {
```

```
    rp <- resolve_pathway(cands)
    pw <- rp$pw
    genes <- rp$genes

    sp <- create_superpathway(pathway_info = pw,
                              celltypes = common_celltypes,
                              gene_sets_celltype = list())
    # assign the gene set to every cell type via the official API
    # (setGeneSetsCelltype with value=NULL pulls from MSigDB internally)
    sp <- setGeneSetsCelltype(sp, gse = gse)
    # keep only genes present in the merged pseudobulk
    sp$gene_sets_celltype <- lapply(sp$gene_sets_celltype,
                                    function(gs) intersect(gs, available_genes))

    hp <- create_hyperparameters(quantile_comb_table = quantile_grid,
                                 outcome_type = "binary",
                                 number_PLS = 2L, folds_CV = 1L, repetition_CV = 1L)

    superpathway_inputs[[pw$standard_name]] <- create_superpathway_input(
        superpathway_info    = sp,
        hyperparameters_info = hp,
        pseudobulk_lognorm   = merged,
        sample_id            = sample_id,
        sample_class         = sample_class,
        base_class           = "HC",
        target_class         = "AD")
}
```

Step 4: Prepare the disease model mapping organism (OXA)

```
load(file.path(extdata_dir, "OXA.rda"))  # OXA (Seurat object)
colnames(slot(OXA, "meta.data"))[c(5, 11, 1)] <- c("class",
                                                   "celltype_cluster",
                                                   "donor")
oxa_org <- create_mapping_organism(
    organism = "Mus musculus",
    target_class = "OXA", base_class = "ETOH",
    celltype_mapping = list(
        "Dendritic Cells" = c("cDC2", "cDC1", "migratory DCs"),
        "Langerhan Cells" = c("LC"),
        "T-cell"          = c("DETC", "T")),
    counts = OXA)
```

Step 5: Fit optimal models, compute recapitulations, and render

```
BiocParallel::register(
    BiocParallel::SnowParam(workers = 2, exportglobals = FALSE,
                            progressbar = FALSE), default = TRUE)

superpathways_fit <- multiple_fitOptimal(
    superpathway_inputs,
    parallel     = rep(TRUE, length(superpathway_inputs)),
    npermut      = 100,
    type         = rep("jackknife", length(superpathway_inputs)),
    nsubsampling = NULL)

BiocParallel::register(BiocParallel::SerialParam(), default = TRUE)

# Requires an active Ensembl connection for mouse-to-human orthology mapping
recapitulations <- multiple_singISTrecapitulations(oxa_org, superpathways_fit)
rendered <- render_multiple_outputs(objects = list(recapitulations))
```

Step 6: Save the .RData files for the Visualizer

```
out_dir <- "extdata"
if (!dir.exists(out_dir)) dir.create(out_dir, recursive = TRUE)
save(superpathways_fit, file = file.path(out_dir, "optimal_models.RData"))
save(rendered,          file = file.path(out_dir, "recapitulations.RData"))
```

Step 7: Launch the Visualizer and load data

```
# Option A: Launch locally
quarto::quarto_run("singIST_visualizer.qmd")

# Option B: Use the hosted version
# https://amoruno.shinyapps.io/singIST_visualizer/
```

Then either upload the two `.RData` files via the upload controls, or click the “Load Example Data” button to use the pre-computed example results (generate them first with `generate_test_data_3pathways.R` if needed).

Quick start: example data only

If you do not need to run the full workflow and just want to explore the Visualizer with pre-computed results:

```
# From the singIST-visualizer repository root
source("generate_test_data_3pathways.R")      # creates extdata/*.RData
quarto::quarto_run("singIST_visualizer.qmd")  # then click "Load Example Data"
```

9. Visual Guide (Screenshots)

This section provides annotated screenshots illustrating each step of the Visualizer workflow. Screenshots are stored in the `figures/` subdirectory of the Visualizer repository.

9.1 Launching the Visualizer

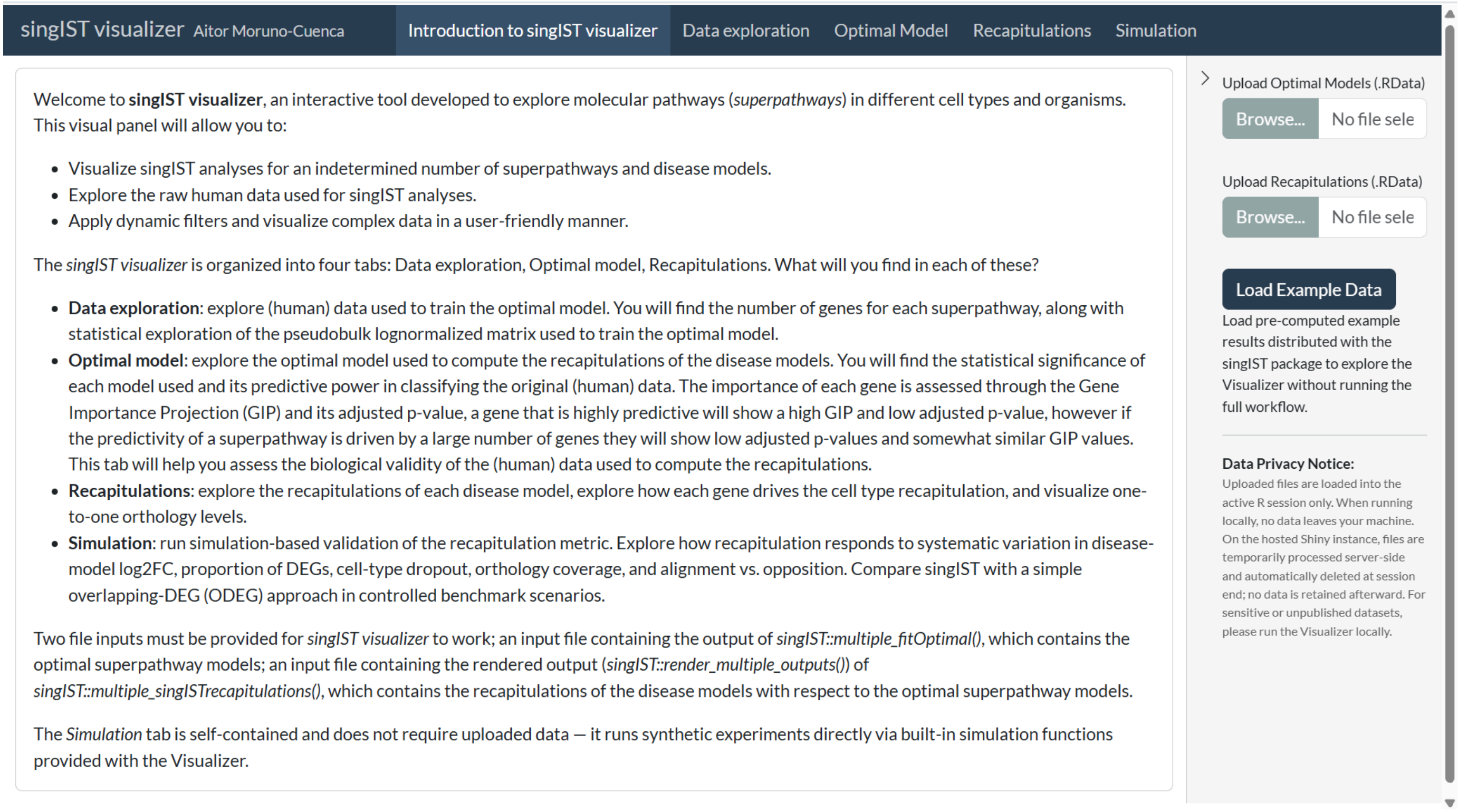

Launching the singIST Visualizer locally from RStudio using quarto::quarto_run().

9.2 Upload page and Load Example Data

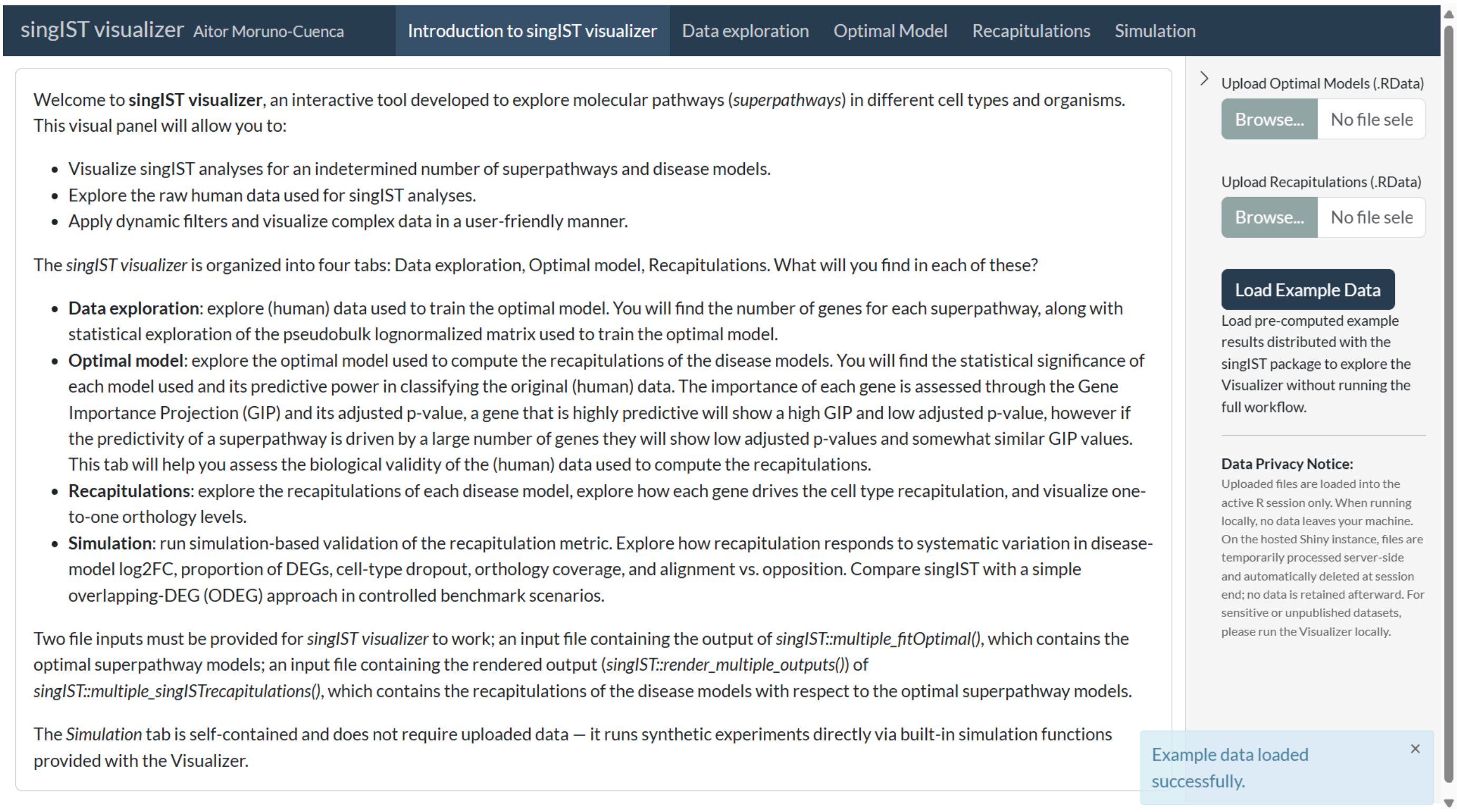

The Visualizer upload page showing the two .RData upload controls and the “Load Example Data” button.

9.3 Unsuccessful upload (error message)

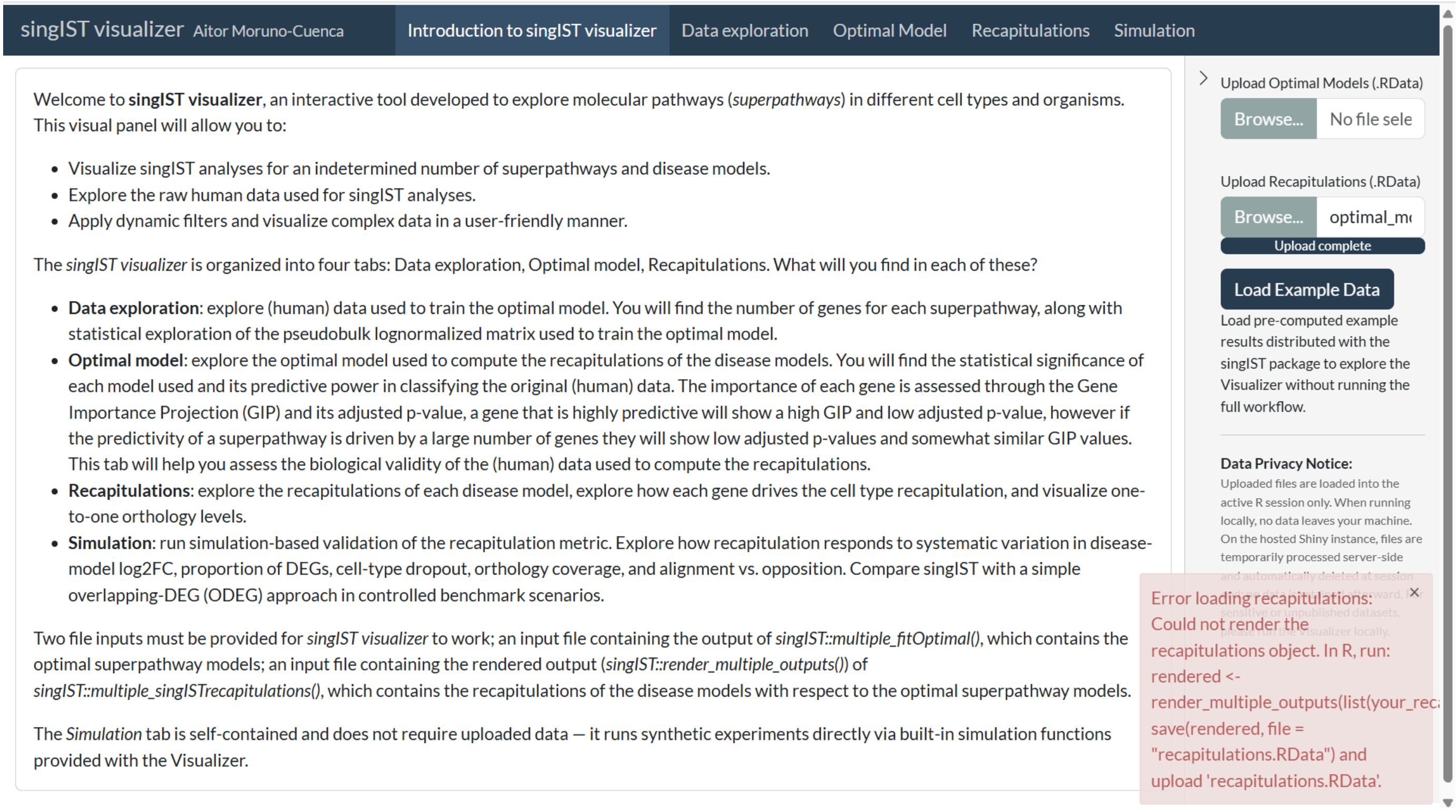

Red error notification shown when an uploaded file does not match the expected singIST structure, with an informative message identifying the problem.

9.4 Data Exploration panel

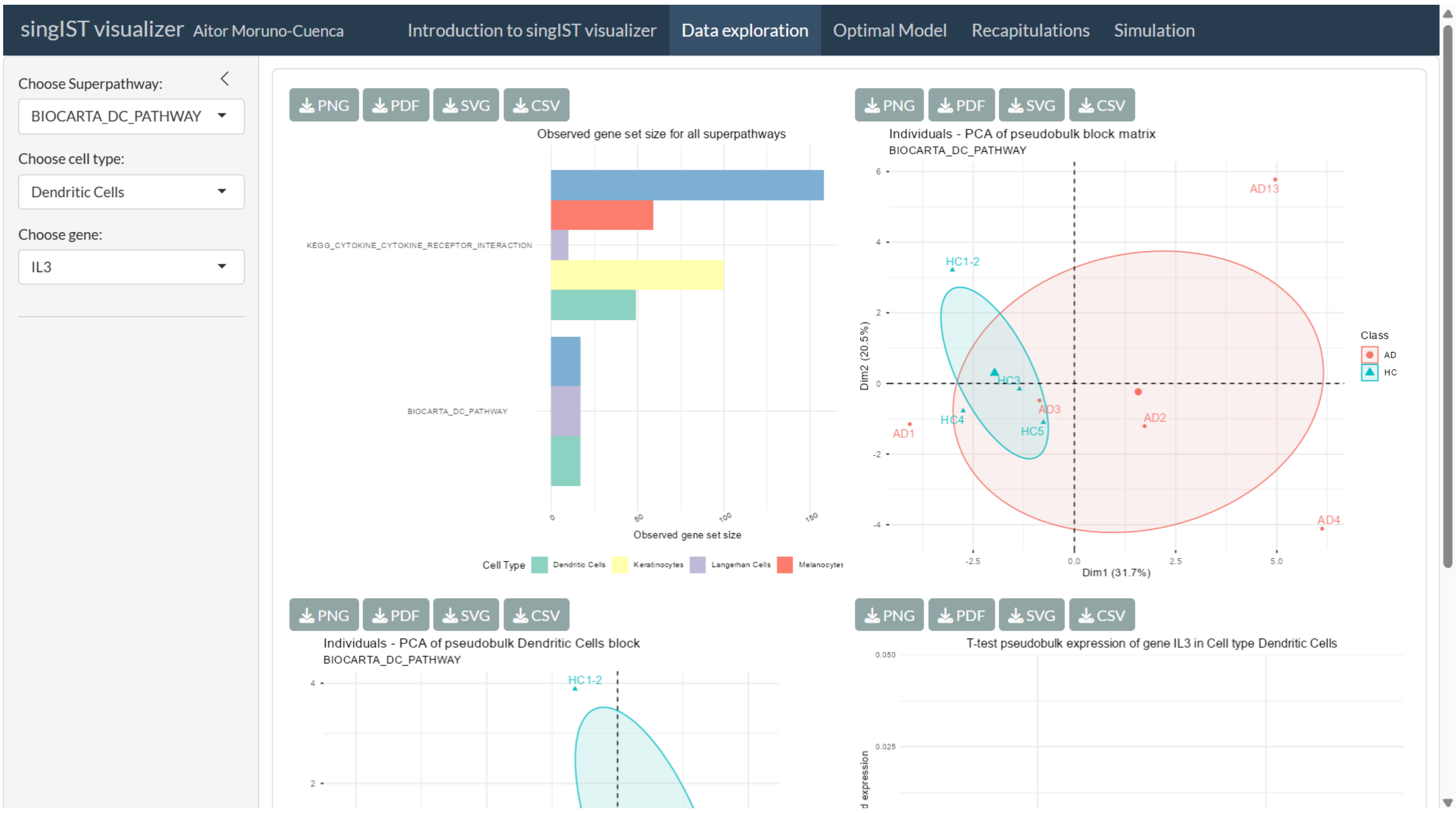

Data Exploration panel: gene-set size bar chart and PCA / expression boxplot for a selected gene and cell type.

9.5 Optimal Model panel

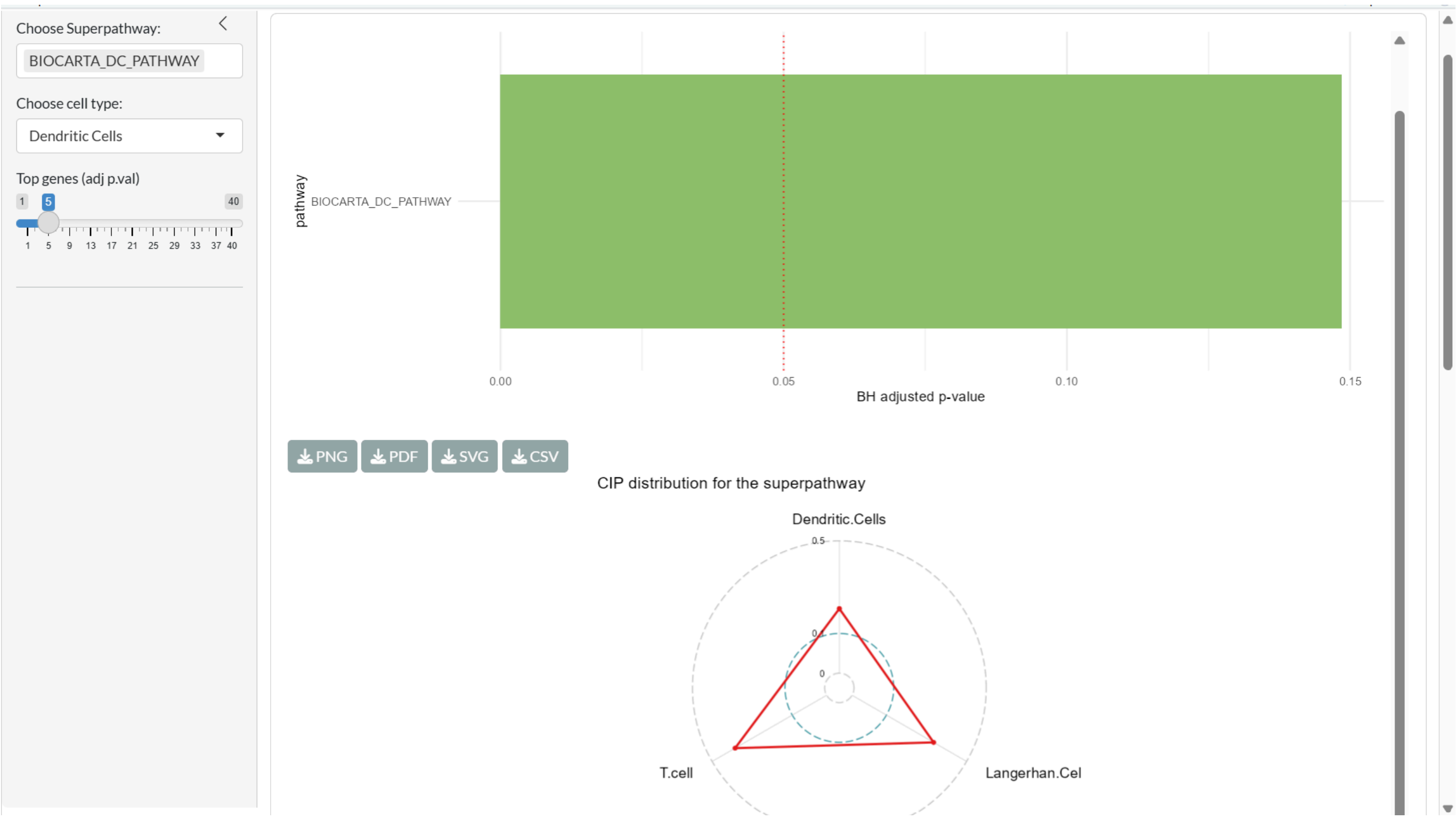

Optimal Model panel: class-separation projection, cell-type contributions, and ranked gene drivers.

9.6 Recapitulations panel

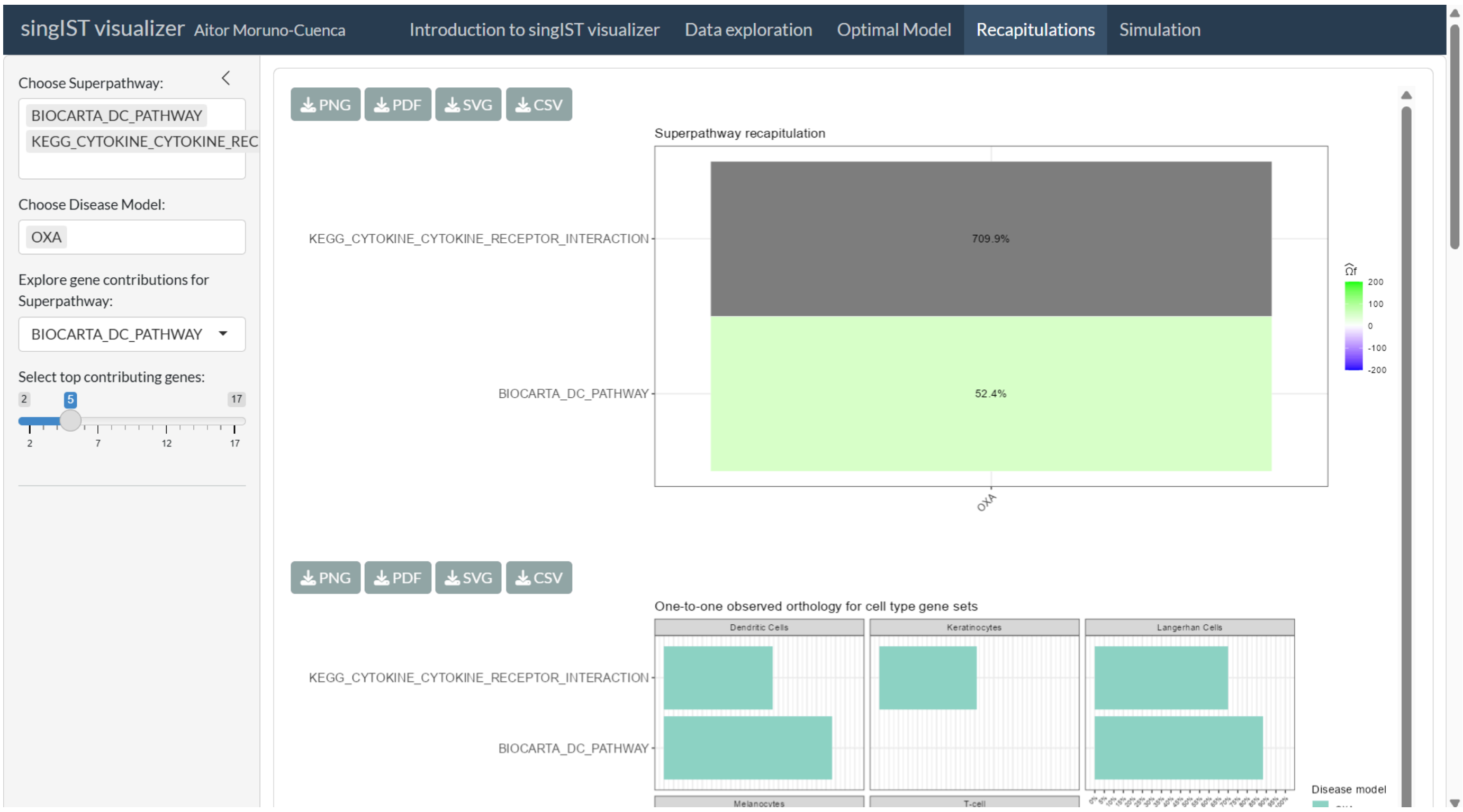

Recapitulations panel: superpathway recapitulation bar chart, cell-type heatmap, and gene-level fold-change scatter plot.

9.7 Simulation panel

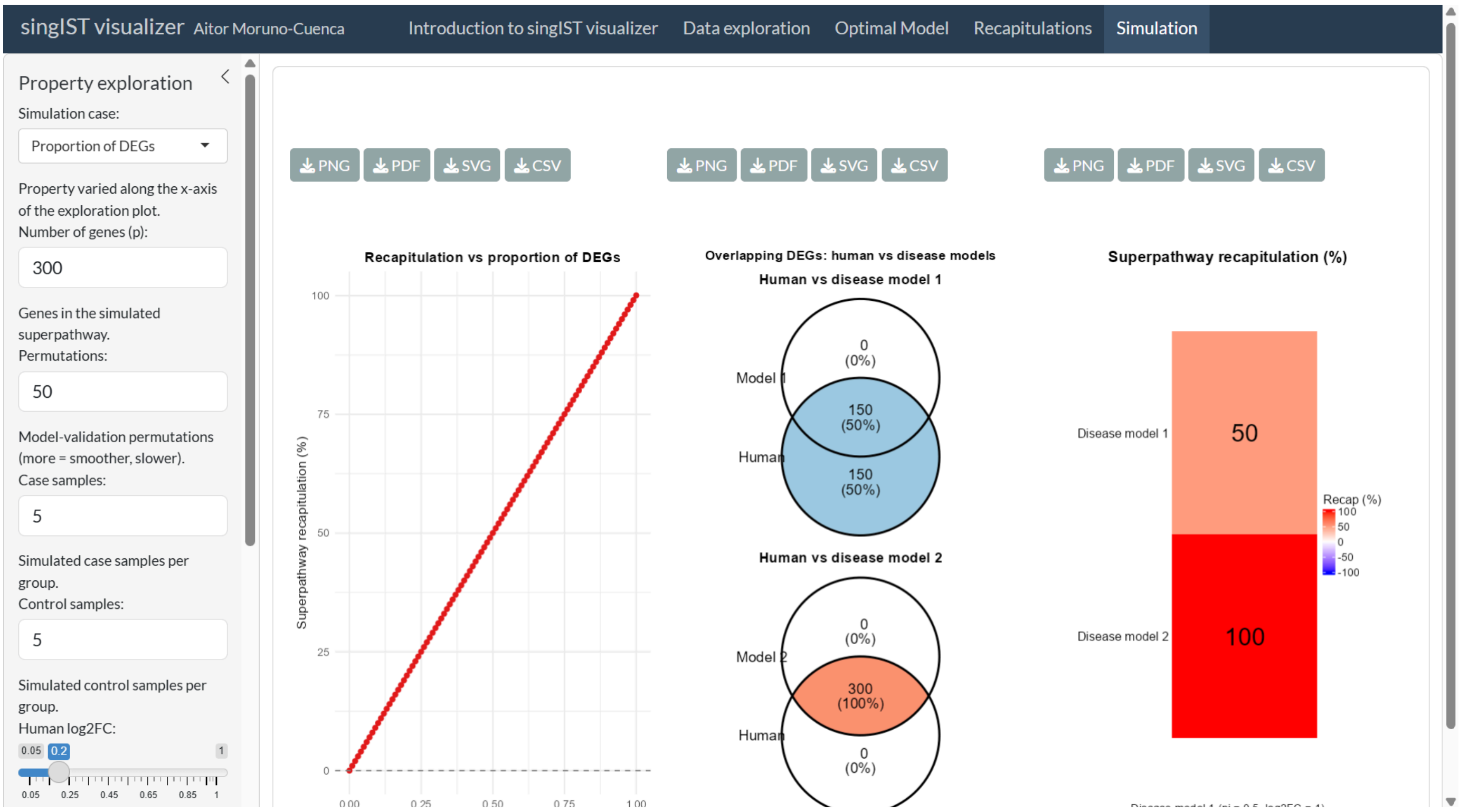

Simulation panel: property-exploration line plot (left) and ODEG-benchmark Venn diagram with singIST recapitulation heatmap (right).

9.8 Exporting figures and tables

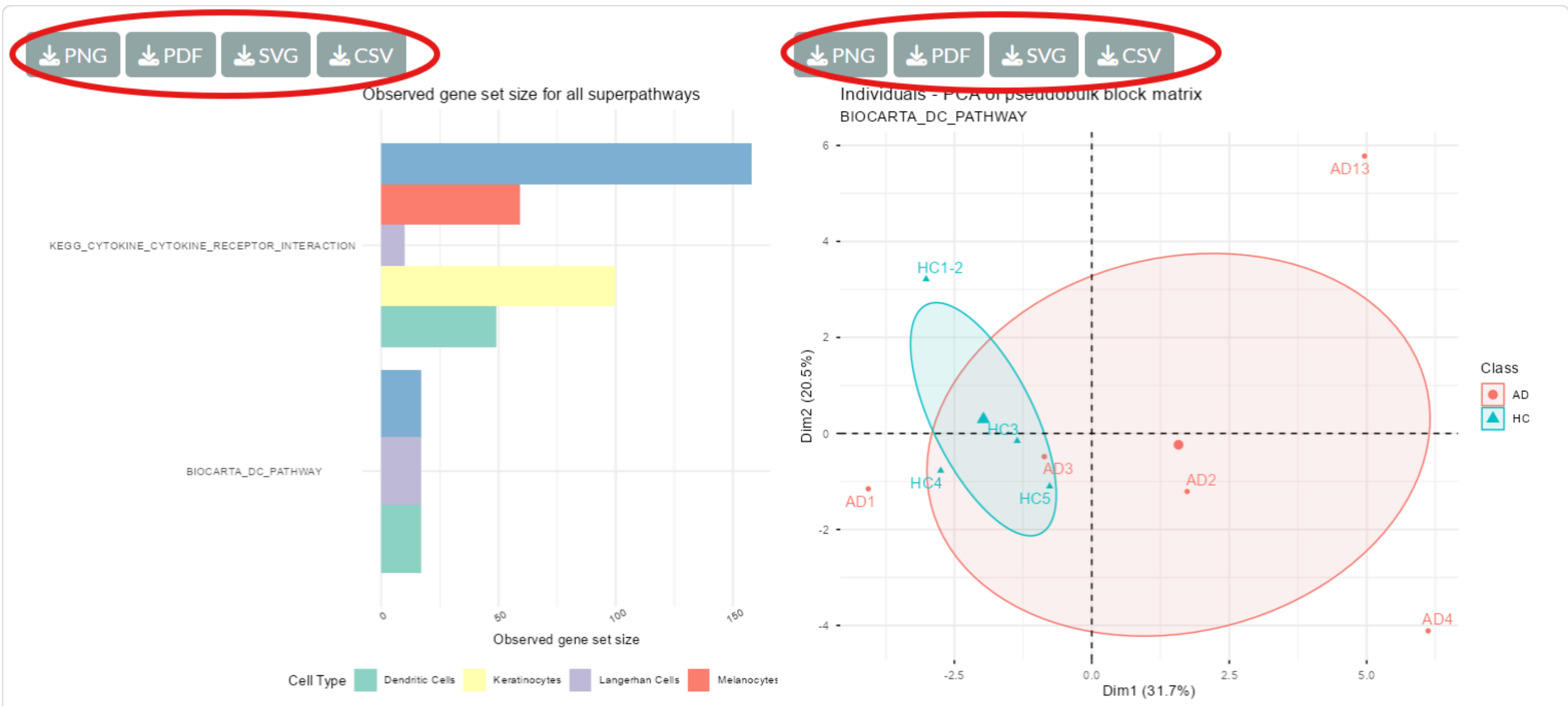

Using the Shiny plot export menu to download a figure as PNG, and the DataTable export buttons to download a table as CSV.

10. Understanding the Simulation Panel

The Simulation panel lets you probe how singIST behaves under controlled conditions, without needing any real dataset. It has two parts: a set of property-exploration cases that show what drives a recapitulation score up or down, and an ODEG benchmark that compares singIST against the most common alternative way of comparing a disease model to a human condition. This section explains, in plain terms, what each part shows and why it matters.

10.1 What a recapitulation score means

A recapitulation score summarizes how well a disease model reproduces a human effect, for a given pathway and cell type. The score is signed:

- Near 100%: the model reproduces the human effect at a comparable magnitude and in the same direction.
- Above 100%: the model reproduces the direction but with a larger effect than the human reference (it "overshoots").
- Below 0%: the model shifts in the opposite direction to the human reference.
- Near 0%: no recapitulation.

Because the score is signed and unbounded, values well below 0% or well above 100% are meaningful (they indicate strong opposition or strong overshoot), not errors. The simulation cases below illustrate how these values arise.

10.2 Property-exploration cases

Each case holds everything fixed except one property of the disease model, so you can see how that single property moves the recapitulation score.

1. Effect size interval. The human effect is fixed and the disease model log2 fold change is varied across an interval. When the model effect is aligned with and close to the human effect, recapitulation sits near 100%; as the model effect grows larger it exceeds 100% (overshoot), and as it crosses zero and reverses sign the score crosses 0% and approaches -100%. This shows that the score tracks both the direction and the magnitude of the effect.

2. Proportion of differentially expressed genes. The fraction of genes declared differentially expressed in the model is varied. Recapitulation scales with this proportion: a model in which more of the pathway genes are actually perturbed recapitulates the human condition better than one in which only a few genes move.

3. Cell-type dropout. Cell types are removed one at a time from the cell-type mapping. Recapitulation decreases toward 0% as cell types are dropped, showing that the score reflects how completely the model's cell-type structure is represented in the comparison.

4. Orthology coverage. The percentage of genes retained after one-to-one orthology mapping is varied from 0 to 100%. Recapitulation scales with coverage, illustrating the direct cost of losing orthologous genes in the cross-species comparison.

5. Alignment vs. opposition of effects. The disease model and human effect directions are set to be aligned or opposed across a range of magnitudes. Aligned effects yield scores centered near 100%; opposed effects yield scores centered near -100%. This is the clearest demonstration that the sign of the score encodes agreement vs. disagreement, not just similarity.

Together, these five cases establish the basic properties a recapitulation metric should satisfy: it should respond to direction, magnitude, the breadth of perturbation, the completeness of the cell-type mapping, and orthology coverage.

10.3 ODEG benchmark: why singIST is compared against overlapping DEGs

In single-cell disease model studies, the most common way to compare a model to the human condition is to compute the overlap of differentially expressed genes (ODEGs) between the model and the human data, often separately for each cell type. A larger overlap is then interpreted as better recapitulation. This is the de facto standard against which a new method should be benchmarked.

The limitation of ODEGs is that the overlap is just a count of shared significant genes. It treats every shared gene as equally important and ignores whether the shared genes move in the same direction or in opposite directions, and it ignores how large the effects are. Two models can therefore produce the same overlap while recapitulating the human condition in very different ways. The benchmark makes this concrete with three scenarios:

1. Agreement. The human and the models differ only in the proportion of genes that are differentially expressed, while direction and magnitude agree. Here ODEGs and singIST agree: both rank the model with the larger overlap as the better recapitulator. This is the easy case where the simple method works.
2. Direction disagreement. The human effect is positive but the models' effects are negative, so the shared DEGs are moving in the opposite direction to the human. ODEGs still favors the model with more overlapping DEGs, because it only counts genes and ignores sign. singIST instead assigns negative recapitulation scores, correctly indicating that neither model actually recapitulates the human condition. This is the key failure mode of the overlap approach.
3. Magnitude disagreement. The human effect has a certain size. One model matches that effect size but perturbs fewer genes; another perturbs more genes but with a much smaller effect. ODEGs prefers the second model (more overlap), whereas singIST gives higher recapitulation to the first model, because its effect sizes match the human reference better. This shows that singIST weights magnitude, not just membership in the significant-gene list.

10.4 Why this matters

The benchmark shows, in an interpretable way, where the standard ODEG approach breaks down and what singIST adds on top of it. singIST integrates all cell types of a pathway into a single model, produces a signed recapitulation score that encodes agreement or disagreement, and decomposes the result into cell-type and gene-level contributions. ODEGs, by contrast, is computed separately per cell type, ignores direction and magnitude, and provides no unified pathway-level measure. The simulation panel lets you explore all of this interactively, and the same cases are available as standalone functions (`run_simulation_case` , `run_odeg_benchmark`) in `simulation_helpers.R` for reproducible, publication-ready figures.
