# Supplementary material for: singIST: an R/bioconductor library and quarto dashboard for automated single-cell comparative transcriptomics analysis of disease models and humans
Source: Front Genet. 2026 Sep 25;17:1952333. doi: 10.3389/fgene.2026.1952333 (PMC13649217; doi:10.3389/fgene.2026.1952333)
Supplement: Supplementary file 3 [file DataSheet3.pdf]

singIST Vignette

Aitor Moruno-Cuenca

21 agosto 2026

Contents

1 Abstract of singIST as a Bioconductor library 2
2 Introduction 2
3 Version information and reproducibility 2
4 Inputs of singIST 3
4.1 Superpathways 3
4.2 Human scRNA-seq 5
4.3 Disease model scRNA-seq 7
5 Fit Optimal asmbPLS-DA and compute validation metrics for the optimal model 9
5.1 Example: fitting cytokine-cytokine receptor interaction and dendritic cells 9
5.2 in regulating 9
6 Compute recapitulation metrics 10
6.1 Example: Evaluating Oxazolone mouse model of Atopic Dermatitis for Dendritic 10
6.2 Cells and cytokine superpathways 10
7 Offline reproducibility 11
8 Appendix: Missing value handling in human pseudobulk matrices 12
8.1 Problem setup 12
8.2 Step 1: Impute the training fold using multiblock MFA. 12
8.3 Step 2: Impute the validation fold by projection (no re-fitting). 13
9 Session Info 13

1 Abstract of singIST as a Bioconductor library

Comparative transcriptomics aims to identify common and differing patterns (organisms, experimental conditions, tissues, and so on) between a set of conditions by using transcriptomics data The methodology to tackle the problem will vary greatly depending on which patterns we aim to compare, the amount and biological nature of them and which omics resolution. **singIST** is a comparative transcriptomics method that aims to compare a disease model against a human disease of interest. In drug discovery and pharmaceutical early development choosing a disease model becomes a fundamental endavour. For this reason, we present **singIST** as a method that compares the single-cell transcriptomics profile of a disease model, either In Vivo or In Vitro models, against a human disease that such model should mimick.

Other Bioconductor packages for comparative transcriptomics are available. One of such is **HybridExpress** which aims to compare the bulk transcriptomics (RNA-seq) profile for hybrids relative to their progenitor species. **CoSIA** is a Bioconductor package that enables organism-to-organism comparative bulk transcriptomics (RNAseq) across tissues. However, none of these packages are direct comparators to **singIST* since they are not conceived for single-cell comparative transcriptomics but for bulk transcriptomics instead.

2 Introduction

This Vignette demonstrates how to use the singIST package. singIST quantifies the direction and mangitude of single-cell transcriptomic changes between a human reference condition and a disease model at pathway, cell type, and gene resolution. Throughout this vignette we will work with the following data:

- "cytokine_counts.rda" Contains Human scRNAseq pseudobulk LogNormalized count matrix. The pseudobulk is aggregated by sample and cell type and contains all genes belonging to the pathway Cytokine-Cytokine receptor interaction [KEGG]
- "DC_counts.rda" Contains Human scRNAseq pseudobulk LogNormalized count matrix. The pseudobulk is aggregated by sample and cell type and contains all genes belonging to the pathway Dendritic Cells in regulating Th1/Th2 Development [BIOCARTA]
- "OXA.rda"

```
set.seed(003)
library(singIST)
```

3 Version information and reproducibility

This vignette is distributed in two places, both generated from the **same source file** (`vignettes/singISTvignette.Rmd`) of the corresponding package release:

- The **Bioconductor rendered HTML** (package landing page), built by the Bioconductor build system from the released package tarball.
- The **GitHub repository source** (`vignettes/singISTvignette.Rmd` in `DataScienceRD-Almirall/singIST`), which reflects the latest development state.

Because the Bioconductor HTML is rebuilt on the Bioconductor build schedule, it can lag behind the GitHub source by one build cycle (typically 1-2 days after a new version is accepted). **Follow the vignette that matches your installed package version**; the versions used to render this document are:

```
version_info <- data.frame(
    field = c("singIST version", "R version"),
    value = c(as.character(utils::packageVersion("singIST")),
              R.version.string))
if (requireNamespace("BiocManager", quietly = TRUE)) {
    version_info <- rbind(version_info, data.frame(
        field = "Bioconductor version",
        value = as.character(BiocManager::version())))
}
version_info
##                  field                                 value
## 1      singIST version                                 1.1.2
## 2            R version R version 4.6.0 (2026-04-24 ucrt)
## 3 Bioconductor version                                  3.23
```

The GitHub source of this vignette corresponds to the installed singIST version (see the version table above). If your installed version differs, check the vignette shipped with your installation via `vignette("singISTvignette", package = "singIST")` or the matching GitHub release tag.

4 Inputs of singIST

The method is defined around three inputs:

- **Superpathways**: pathway gene sets structured by cell type.
- **Human scRNA-seq reference (pseudobulk)**: transformed into cell-type specific pseudobulk, restricted to the genes of the superpathway.
- **Disease model scRNA-seq**: a Seurat or SingleCellExperiment object containing scRNA-seq readouts of the disease model to assess.

In the following subsections we explicitly construct these inputs. The rest of the vignette uses these inputs to: (1) learn a human reference model using adaptive sparse multi-block PLS-DA, and then (2) evaluate how human-like the disease model changes are, in the learned human reference space.

4.1 Superpathways

singIST does not operate on a pathway gene set alone. Instead, it defines a superpathway by three things:

- A curated **pathway** (e.g., an MSigDB pathway identifier).
- A set of **cell types** of interest that will be modeled explicitly.
- A **gene set per cell type**, where each cell type is associated with a vector of genes that might or might not be the same

In many practical cases, we use the same pathway gene set for every cell type. However, singIST also supports **cell-type specific gene sets**. Below we create two example of superpathways used later in this vignette.

First, because singIST relies on MSigDB curated pathways, we load the MSigDB gene set collection once and reuse it as necessary. We assume the reference to compare against is human `org = "hs"` and we extract both ENTREZID and HGNC gene symbols `id = c("SYM", "EZID")`.

```
gse <- msigdb::getMsigdb(org = "hs", id = c("SYM", "EZID"),
                         version = msigdb::getMsigdbVersions()[1])
```

4.1.1 Example 1: Superpathway with the same pathway gene set for each cell type

In this first example, we define a pathway using its MSigDB metadata, choose the cell types we want to model, and then assign the same pathway gene set to each cell type gene set. This is the simplest and most common setup for a superpathway.

```
# Pathway definition (curated gene set identifier)
dc_pathway <- create_pathway(standard_name = "BIOCARTA_DC_PATHWAY",
                             dbsource = "BIOCARTA",
                             collection = "c2",
                             subcollection = "CP")
# Superpathway = pathway + modeled cell types + per cell type gene sets
dc_superpathway <- create_superpathway(pathway_info = dc_pathway,
                                       celltypes = c("Dendritic Cells",
                                                     "Langerhan Cells",
                                                     "T-cell"),
                                       list())
# Assign gene sets per cell type (retrieve gene set from MSigDB)
dc_superpathway <- setGeneSetsCelltype(dc_superpathway, gse = gse)
```

4.1.2 Example 2: Superpathway with different gene sets per cell type (optional)

In some applications, you may want different cell types to use different gene sets. singIST supports this by allowing the user to directly provide a list of gene vectors, one per cell type.

Here we demonstrate this capability by taking the curated pathway gene set and creating different subsets for each cell type. (This is purely illustrative; in real analyses these subsets would be defined biologically rather than randomly sampled.)

```
# Pathway definition (curated gene set identifier)
cytokine_pathway <- create_pathway(
    standard_name = "KEGG_CYTOKINE_CYTOKINE_RECEPTOR_INTERACTION",
    dbsource = "KEGG",
    collection = "c2",
    subcollection = "CP"
)
# Superpathway = pathway + modeled cell types + per cell type gene sets
cytokine_superpathway <- create_superpathway(
    pathway_info = cytokine_pathway,
    celltypes = c("Dendritic Cells", "Keratinocytes", "Langerhan Cells",
                  "Melanocytes", "T-cell"),
    list()
)
```

```r
# Pull the curated gene set (retrieve gene set from MSigDB)
gene_set <- pullGeneSet(cytokine_pathway, gse = gse)
# Provide a different gene set per cell type
cytokine_superpathway$gene_sets_celltype <- list(
    "Dendritic Cells" = sample(gene_set, 50),
    "Keratinocytes" = sample(gene_set, 100),
    "Langerhan Cells" = sample(gene_set, 10),
    "Melanocytes" = sample(gene_set, 60),
    "T-cell" = sample(gene_set, 160)
)
```

4.2 Human scRNA-seq

The human reference data are used to train an adaptive sparse multi-block PLS on a superpathway. In this subsection we start from the human scRNA-seq that have already been aggregated into cell-type pseudobulk log-normalize expression. For instance, the pseudobulk can be performed with `Seurat::AggregateExpression()`. Concretely the pseudobulk matrix:

- Each row corresponds to a pseudobulk profile for a cell type within a sample (e.g., T-cell_AD1).
- Columns correspond to genes (typically HGNC symbols for human training).
- Values correspond to log-normalized pseudobulk expression.

In addition to the pseudobulk matrix, singIST requires sample level labels defining a base class and a target class (e.g., HC vs AD). The target class is the human experimental group that the disease model such mimic precisely, while the base class is the human experimental group that such differentiate from the target class.

The training procedure includes a tuning step, so the human input is not only the pseudobulk matrix and sample labels - it also includes the hyperparameter search space and the cross-validation design the user wants. In practice, we tune:

- Block-specific sparsity via a grid of quantile thresholds (one quantile per modeled cell type). Intuitively, higher quantiles enforce stronger sparsity (fewer genes retained) in that block.
- Model complexity via `number_PLS` (maximum number of latent components to test).
- Validation scheme via `folds_CV` and `repetition_CV`.

We store the former settings in a `hyperparameters` list and then combine all the information into the `superpathway_input` list, which is the main training input consumed by the training procedure `fitOptimal()/ multiple_fitOptimal()`.

4.2.1 Example 1: Dendritic Cells in regulating Th1/Th2 Development

```r
# Load the pseudobulk matrix
file_dc <- system.file("extdata", "DC_counts.rda", package = "singIST")
obj_dc <- load(file_dc)
counts_dc <- get(obj_dc[1])
# Remove the cell types that we are not going to model
counts_dc <- counts_dc[!(sub("_.*$", "", rownames(counts_dc)) %in%
                         c("MastC-other", "Keratinocytes", "Melanocytes")), ]
```

Next, we define the sample identifiers and class labels.

```
# Load the pseudobulk matrix
sample_id_dc <- c("AD1", "AD13", "AD2", "AD3","AD4", "HC1-2", "HC3", "HC4",
                  "HC5")
sample_class_dc <- c(rep("AD", 5), rep("HC", 4))
base_class_dc <- "HC"
target_class_dc <- "AD"
```

Now we define the hyperparameters.

```
# Initialize hyperparameter object
# Choose the array of quantile combinations that will be tested in the Cross
# Validation. Ideally one should choose a wide range of quantiles to test,
# as a narrow selection might not find the globally optimal model. The more
# quantiles one chooses the more computation time it will take, for the sake
# of simplicity we choose a small amount of quantiles to test
quantile_comb_table_dc <- as.matrix(
    RcppAlgos::permuteGeneral(seq(0.1, 0.95, by = 0.3),
                              m = length(dc_superpathway$celltypes),
                              TRUE))
# Store tuning + CV design
dc_hyperparameters <- create_hyperparameters(
    quantile_comb_table = quantile_comb_table_dc,
    outcome_type = "binary",
    number_PLS = as.integer(3),
    folds_CV = as.integer(1), #LOOCV
    repetition_CV = as.integer(1)
)
```

Finally, we combine all the information into a superpathway_input list.

```
dc_superpathway_input <- create_superpathway_input(
    superpathway_info = dc_superpathway,
    hyperparameters_info = dc_hyperparameters,
    pseudobulk_lognorm = counts_dc,
    sample_id = sample_id_dc,
    sample_class = sample_class_dc,
    base_class = base_class_dc,
    target_class = target_class_dc
)
```

4.2.2 Example 2: Cytokine-Cytokine receptor interaction superpathway

```
# Load the pseudobulk matrix
file_cytokine <- system.file("extdata", "cytokine_counts.rda", package = "singIST")
obj_cytokine <- load(file_cytokine)
counts_cytokine <- get(obj_cytokine[1])
# Remove the cell types that we are not going to model
counts_cytokine <- counts_cytokine[!(sub("_.*$", "", rownames(counts_cytokine))
                                     %in%
                                 c("MastC-other")), ]
```

Next, we define the sample identifiers and class labels.

```
# Load the pseudobulk matrix
sample_id_cytokine <- c("AD1", "AD13", "AD2", "AD3","AD4", "HC1-2", "HC3",
                        "HC4", "HC5")
sample_class_cytokine <- c(rep("AD", 5), rep("HC", 4))
base_class_cytokine <- "HC"
target_class_cytokine <- "AD"
```

Now we define the hyperparameters.

```
# Initialize hyperparameter object
quantile_comb_table <- as.matrix(
    RcppAlgos::permuteGeneral(seq(0.05, 0.95, by = 0.2),
                            m = length(cytokine_superpathway$celltypes),
                            TRUE))
# Store tuning + CV design
cytokine_hyperparameters <- create_hyperparameters(
                                quantile_comb_table = quantile_comb_table,
                                outcome_type = "binary",
                                number_PLS = as.integer(2),
                                folds_CV = as.integer(5),
                                repetition_CV = as.integer(5))
```

Finally, we combine all the information into a superpathway_input list.

```
cytokine_superpathway_input <- create_superpathway_input(
    superpathway_info = cytokine_superpathway,
    hyperparameters_info = cytokine_hyperparameters,
    pseudobulk_lognorm = counts_cytokine,
    sample_id = sample_id_cytokine,
    sample_class = sample_class_cytokine,
    base_class = base_class_cytokine,
    target_class = target_class_cytokine)
```

4.3 Disease model scRNA-seq

The disease model input is a single-cell object (either a `Seurat` or `SingleCellExperiment`) containing scRNA-seq measurements from the model system we want to assess. Unlike the human input (which is provided as pseudobulk matrices), the disease model is kept at single-cell resolution and is summarized internally by singIST when computing recapitulation scores.

To use a disease model dataset, singIST needs:

- A class label indicating the base and target experimental groups in the model (e.g. ETOH vs OXA).
- A cell-type cluster label used to map model cell populations to the human cell types defined in the superpathway.
- A donor / sample identifier.

Finally, we must provide a cell type mapping from th ehuman superpathway cell types (names used in `celltypes = ...`) to the disease model clusters. A human cell type can map to one or more model clusters. A human cell type can map to one or more model clusters; it is also valid to provide an empty mapping (meaning that block will not contribute for that model).

In the following example we load the oxazolone mouse dataset shipped in the package, standarize metadata column names to those expected by singIST, define the mapping, and construct a `mapping organism` list via `create_mapping_organism()`.

4.3.1 Example: Oxazolone mouse model of Atopic Dermatitis

```
file_oxa <- system.file("extdata", "OXA.rda", package = "singIST")
obj_oxa <- load(file_oxa)
oxazolone <- get(obj_oxa[1])
```

Next, we ensure the object contains the metadata fields required by singIST. The package example dataset already contains the necessary information, but we rename the relevant columns to the standarized names used downstream: `class`, `celltype_cluster`, and `donor`.

```
# The example object included in singIST uses these column positions
colnames(slot(oxazolone, "meta.data"))[c(5,11,1)] <- c("class",
                                                        "celltype_cluster",
                                                        "donor")
# If the object is a SingleCellExperiment, metadata live in colData()
```

Now, we define the human to model cell type mapping. The names of the list must match the human cell types used in our superpathways. Values correspond to one or more model clusters present in `celltype_cluster`.

```
celltype_mapping <- list(
                    "Dendritic Cells" = c("cDC2", "cDC1", "migratory DCs"),
                    "Keratinocytes" = c("Keratinocytes"),
                    "Langerhan Cells" = c("LC"),
                    "Melanocytes" = c(),
                    "T-cell" = c("DETC", "T"))
```

Finally, we combine the model object and mapping into a `mapping_organism` list. This list specifies the model organism we are working with, and which class labels correspond to the base and target conditions.

```
oxa_org <- create_mapping_organism(organism = "Mus musculus",
                target_class = "OXA", base_class = "ETOH",
                celltype_mapping = list(
                    "Dendritic Cells" = c("cDC2", "cDC1", "migratory DCs"),
                    "Keratinocytes" = c("Keratinocytes"),
                    "Langerhan Cells" = c("LC"),
                    "Melanocytes" = c(),
                    "T-cell" = c("DETC", "T")),
                counts = oxazolone)
```

5 Fit Optimal asmbPLS-DA and compute validation metrics for the optimal model

At this stage we have constructed two key training inputs: `dc_superpathway_input` and `cytokine_superpathway_input`. The next step is to train an adaptive sparse multi-block PLS-DA model (asmbPLS-DA) for each superpathway, while selecting an optimal configuration (sparsity per cell type block and number of PLS components) under the cross-validation design specified in the corresponding `hyperparameters` object.

singIST provides two entry points to do it:

- `fitOptimal()` fits one `superpathway_input` list.
- `multiple_fitOptimal()` is a convenience wrapper that fits multiple superpathways in one call.

If the CV design is a leave-one-out CV (LOOCV), the functions provide with a parallelization option to make the computations faster via using `parallel = TRUE` and setting the desired paralellization scheme with `BiocParallel::register()`.

In addition to fitting the optimal model, singIST can compute model validation metrics, including permutation based global significance test (`npermut = 100`) and resampling permutation tests (`type`, e.g., jackknife or subsampling). The number of permutation for the tests can be chosen as desired.

5.1 Example: fitting cytokine-cytokine receptor interaction and dendritic cells

5.2 in regulating

If the human pseudobulk input contains missing values (e.g., a cell type block absent for some samples), `fitOptimal() / multiple_fitOptimal()` automatically imputes them during model fitting. Further details of the imputation procedure are provided in the Appendix.

```
# Register a parallel backend (SnowParam works on Windows/macOS/Linux);
# on Linux you may prefer MulticoreParam.
BiocParallel::register(BiocParallel::SnowParam(workers = 2,
                        exportglobals = FALSE, progressbar = FALSE),
                        default = TRUE)
# Combine all human training inputs into a list
superpathways <- list(dc_superpathway_input,
                      cytokine_superpathway_input)
# Fit Optimal asmbPLS-DA models + compute validation metrics
# parallel: logical vector indicating whether the CV design should be parallel
# npermut: number of permutations used for permutation based tests
# type : validation strategy (jackknife or subsampling)
# nsubsampling: number of subsampling repeats (only used when type includes
# subsampling).
superpathways_fit <- multiple_fitOptimal(
    superpathways,
    parallel = c(TRUE, FALSE),
    npermut = c(100),
    type = c("jackknife","subsampling"),
```

```
    nsubsampling = c(NULL, 5)
    )
# Restore serial backend (good practice)
BiocParallel::register(BiocParallel::SerialParam(), default = TRUE)
```

6 Compute recapitulation metrics

After fitting the optimal human reference models (`superpathway_fit`), the final step is to quantify how well a disease model recapitulates the human target vs base signal for each superpathway. singIST does this by applying the disease model changes to the human base class reference and then evaluating how "target-like" the samples become. The resulting recapitulation outputs can be summarized at pathway, cell type, and gene resolution.

There are two functions to do this:

- `singISTrecapitulations()` computes recapitulations for one fitted superpathway model.
- `multiple_singISTrecapitulations()` is a wrapper that computes recapitulations for multiple fitted models at once.

Below we compute recapitulations for both fitted superpathways against the oxazolone mapping organism `oxa_org`, and then render the results into a plotting friendly output

6.1 Example: Evaluating Oxazolone mouse model of Atopic Dermatitis for Dendritic

6.2 Cells and cytokine superpathways

By default, the function assumes the organism for which the asmbPLS-DA model was trained is "hsapiens", it also assumes that the gene annotation for the mapped organism is "external_gene_name". These parameters are by default but other cases can also be considered by specifying the parameters `from_to = "hsapiens"` and `object_gene_identifiers = "external_gene_name"`.

```
# Compute singIST recapitulations for all fitted superpathways.
# This step queries Ensembl BioMart for one-to-one orthologs. If Ensembl is
# temporarily unavailable, we fall back to the precomputed orthology mapping
# shipped with the package so that the vignette remains fully reproducible
# offline (see section "Offline reproducibility" below).
recapitulations_multiple <- tryCatch(
    multiple_singISTrecapitulations(oxa_org, superpathways_fit),
    error = function(e) {
        message("Live Ensembl BioMart query failed: ", conditionMessage(e),
                "Falling back to the cached orthology mapping shipped ",
                "with singIST.")
        load(system.file("extdata", "example_orthology_mapping.rda",
                         package = "singIST"))
        multiple_singISTrecapitulations(
            oxa_org, superpathways_fit,
            orthology_cache = example_orthology_mapping)
```

```
    }
)
```

In many workflows you will want the outputs in a standardized format that is convenient for downstream plotting and reporting. The helper function `render_multiple_outputs()` reshapes the recapitulation objects accordingly. It makes a list that is ready to plot.

```
final_output <- render_multiple_outputs(objects= list(recapitulations_multiple))
```

7 Offline reproducibility

The only step of the singIST workflow that requires an external service is the **one-to-one orthology mapping** performed by `orthology_mapping()`, which queries Ensembl BioMart. Ensembl occasionally experiences service interruptions or platform migrations: when that happens, older package versions failed with opaque errors such as `HTTP 404 Not Found`.

From this version of singIST onwards:

1. **Informative error messages.** If an Ensembl BioMart request fails, the error states exactly which step and dataset failed (connection, ortholog retrieval, or identifier mapping) and what actions you can take: retry later, let the automatic mirror fallback (`www`, `useast`, `asia`) handle transient failures, connect to an Ensembl archive release host, or provide a precomputed mapping.
2. **Cached orthology mappings.** All recapitulation functions `singISTrecapitulations()` and `multiple_singISTrecapitulations()` accept an `orthology_cache` argument. When provided, no Ensembl queries are performed and the workflow runs fully offline. The cache is a `data.frame` with at least the columns `input_gene` (reference organism identifier, e.g. human gene symbol) and `output_gene` (mapped organism identifier, matching the gene names of the disease model object).

An example human-to-mouse cache (17,066 one-to-one orthologous gene pairs, gene symbols and Ensembl IDs) is shipped with the package:

```
# Load the cached one-to-one orthology mapping shipped with singIST
load(system.file("extdata", "example_orthology_mapping.rda",
                 package = "singIST"))
head(example_orthology_mapping)
##   input_gene output_gene   input_ensembl     output_ensembl
## 1      GNAI3       Gnai3 ENSG00000065135 ENSMUSG00000000001
## 2      CDC45       Cdc45 ENSG00000093009 ENSMUSG00000000028
## 3      SCML2       Scml2 ENSG00000102098 ENSMUSG00000000037
## 4       APOH        Apoh ENSG00000091583 ENSMUSG00000000049
## 5       NARF        Narf ENSG00000141562 ENSMUSG00000000056
## 6       CAV2        Cav2 ENSG00000105971 ENSMUSG00000000058

# Run the recapitulation step fully offline
recapitulations_offline <- multiple_singISTrecapitulations(
    oxa_org, superpathways_fit,
    orthology_cache = example_orthology_mapping)
```

To build a cache for your own organism pair, query Ensembl BioMart once while the service is available and save the result:

```
# Example: build your own human-to-mouse one-to-one orthology cache
mart_hs <- biomaRt::useEnsembl(biomart = "genes",
                               dataset = "hsapiens_gene_ensembl")
my_cache <- biomaRt::getBM(
    attributes = c("external_gene_name", "ensembl_gene_id",
                   "mmusculus_homolog_associated_gene_name",
                   "mmusculus_homolog_ensembl_gene",
                   "mmusculus_homolog_orthology_type"),
    mart = mart_hs)
my_cache <- my_cache[my_cache$mmusculus_homolog_orthology_type ==
                         "ortholog_one2one", ]
colnames(my_cache) <- c("input_gene", "input_ensembl", "output_gene",
                        "output_ensembl", "orthology_type")
save(my_cache, file = "my_orthology_cache.rda")
```

Genes of a superpathway gene set without a one-to-one ortholog in the cache are excluded from the mapping (with a warning reporting how many), exactly as they would be if no one-to-one ortholog were found in Ensembl, so orthology coverage statistics remain correct.

8 Appendix: Missing value handling in human pseudobulk matrices

During CV, singIST may encounter missing entries in the human pseudobulk predictor (e.g., entire cell type blocks missing for some samples). Rather than discarding those samples, singIST imputes missing values inside each CV split in a way that preserves the multiblock structure and avoids information leakage. This procedure is performed automatically by `fitOptimal()` and `multiple_fitOptimal()` and the user does not provide any input on this, this section is merely for informative purposes.

8.1 Problem setup

Assume we have a multi-block dataset for which we aim to impute an asmbPLS-DA, $D = \{(x_i, y_i)\}_{i=1}^{n}$ where $x_i \in \mathbb{R}^p, y_i \in \{0,1\}$. We perform a K-fold CV, thus splitting the dataset into K folds; $\mathcal{T}_k$ training indices, $\mathcal{V}_k$ validation indices, $k = 1, \dots, K$. Then we have a classic train-test dataset $\left(X_{train}^{(k)}, Y_{train}^{(k)}\right) \in \{(x_i, y_i) : i \in \mathcal{T}_k\}$ and $\left(X_{val}^{(k)}, Y_{val}^{(k)}\right) \in \{(x_i, y_i) : i \in \mathcal{V}_k\}$

The problem lies in that X matrices we might have missing values.

8.2 Step 1: Impute the training fold using multiblock MFA.

We first impute $X_{train}^{(k)}$ using Multple Factor Analysis (MFA) imputation (via `missMDA::imputeMFA()`), which explicitly accounts for the multiblock structure (blocks correspond to modeled cell types). This produces an imputed training matrix $\widetilde{X}_{train}^{(k)}$.

8.3 Step 2: Impute the validation fold by projection (no re-fitting).

To avoid data leakage, we do not refit the MFA model on the validation data. Instead, we compute MFA loadings and means from the imputed training fold and project each validation sample using its observed features only.

Let $\mu \in \mathbb{R}^p$ be the vector of variable means and $P \in \mathbb{R}^{p \times ncp}$ the matrix of MFA loadings estimated from $\widetilde{X}^{(k)}_{train}$. For each validation sample $x_i \in X^{(k)}_{val}$ define the set of observed feature indices:

$$O_i = \{j : (x_i)_j \neq NA\}, \quad |O_i| \geq 1$$

We estimate the latent scores t_i by solving a least-squares problem restricted to the observed features:

$$t_i = \arg\min_{u_i \in \mathbb{R}^{ncp}} ||x_{i,O_i} - (\mu_{O_i} + P_{O_i} u_i)||_2^2 = \left(P_{O_i}^T P_{O_i}\right)^{-1} P_{O_i}^T \left(x_{i,O_i} - \mu_{O_i}\right)$$

We then reconstruct the feature full vector by:

$$\widehat{x}_{ij} = \mu_j + P_{j \cdot} t_i$$

The imputation validation entries are defined as:

$$\widehat{x}_{ij} = \begin{cases} x_{ij}, & j \in O_i \\ \widetilde{x}_{ij}, & j \notin O_i \end{cases} \quad i \in \mathcal{V}_k$$

This preserves the same latent space used for training-fold imputation and prevents leakage from validation into the imputation model. Imputation is intended for **partial missingness** (e.g., one missing block while other blocks are observed). If missingness is extensive (e.g., many samples missing most blocks), model stability and interpretability may degrade; in that case consider restricting the set of modeled cell types and/or increasing sample size.

9 Session Info

```
utils::sessionInfo()
## R version 4.6.0 (2026-04-24 ucrt)
## Platform: x86_64-w64-mingw32/x64
## Running under: Windows 11 x64 (build 26100)
##
## Matrix products: default
##   LAPACK version 3.12.1
##
## locale:
## [1] LC_COLLATE=Spanish_Spain.utf8  LC_CTYPE=Spanish_Spain.utf8
## [3] LC_MONETARY=Spanish_Spain.utf8 LC_NUMERIC=C
## [5] LC_TIME=Spanish_Spain.utf8
##
## time zone: Europe/Madrid
```

```
## tzcode source: internal
##
## attached base packages:
## [1] stats4    stats     graphics  grDevices utils     datasets  methods
## [8] base
##
## other attached packages:
##  [1] GSEABase_1.74.0      graph_1.90.0         annotate_1.90.0
##  [4] XML_3.99-0.23        AnnotationDbi_1.74.0 IRanges_2.46.0
##  [7] S4Vectors_0.50.1     Biobase_2.72.0       BiocGenerics_0.58.1
## [10] generics_0.1.4       msigdb_1.20.0        BiocStyle_2.40.0
## [13] singIST_1.1.2        testthat_3.3.2       devtools_2.5.2
## [16] usethis_3.2.1
##
## loaded via a namespace (and not attached):
##   [1] progress_1.2.3              nnet_7.3-20
##   [3] goftest_1.2-3               DT_0.34.0
##   [5] Biostrings_2.80.1           vctrs_0.7.3
##   [7] spatstat.random_3.5-1       digest_0.6.39
##   [9] png_0.1-9                   shape_1.4.6.1
##  [11] ggrepel_0.9.8               deldir_2.0-4
##  [13] parallelly_1.48.0           MASS_7.3-65
##  [15] reshape2_1.4.5              httpuv_1.6.17
##  [17] foreach_1.5.2               withr_3.0.3
##  [19] showtextdb_3.0              xfun_0.60
##  [21] ggpubr_1.0.0                ellipsis_0.3.3
##  [23] survival_3.8-6              memoise_2.0.1
##  [25] emmeans_2.0.4               Seqinfo_1.2.0
##  [27] gmp_0.7-5.1                 sysfonts_0.8.9
##  [29] zoo_1.9-0                   pbapply_1.7-4
##  [31] Formula_1.2-6               prettyunits_1.2.0
##  [33] KEGGREST_1.52.2             promises_1.5.0
##  [35] otel_0.2.0                  scatterplot3d_0.3-45
##  [37] httr_1.4.8                  rstatix_1.1.0
##  [39] globals_0.19.1              fitdistrplus_1.2-6
##  [41] rstudioapi_0.19.0           pan_2.0
##  [43] miniUI_0.1.2                curl_7.1.0
##  [45] ScaledMatrix_1.20.0         polyclip_1.10-7
##  [47] ExperimentHub_3.2.2         SparseArray_1.12.2
##  [49] FactoMineR_2.16             xtable_1.8-8
##  [51] stringr_1.6.0               desc_1.4.3
##  [53] doParallel_1.0.17           evaluate_1.0.5
##  [55] S4Arrays_1.12.0             BiocFileCache_3.2.0
##  [57] hms_1.1.4                   glmnet_5.0
##  [59] GenomicRanges_1.64.0        bookdown_0.47
##  [61] irlba_2.3.7                 filelock_1.0.3
##  [63] ROCR_1.0-12                 reticulate_1.46.0
##  [65] spatstat.data_3.1-9         magrittr_2.0.5
##  [67] lmtest_0.9-40               later_1.4.8
##  [69] lattice_0.22-9              spatstat.geom_3.8-2
##  [71] future.apply_1.20.2         scattermore_1.2
```

```
##  [73] scuttle_1.22.0             cowplot_1.2.0
##  [75] matrixStats_1.5.0          RcppAnnoy_0.0.23
##  [77] pillar_1.11.1              nlme_3.1-169
##  [79] iterators_1.0.14           compiler_4.6.0
##  [81] beachmat_2.28.0            RSpectra_0.16-2
##  [83] stringi_1.8.9              showtext_0.9-8
##  [85] jomo_2.7-6                 tensor_1.5.1
##  [87] minqa_1.2.8                SummarizedExperiment_1.42.0
##  [89] missMDA_1.22               plyr_1.8.9
##  [91] crayon_1.5.3               abind_1.4-8
##  [93] ggtext_0.1.2               locfit_1.5-9.12
##  [95] sp_2.2-3                   org.Hs.eg.db_3.23.1
##  [97] bit_4.6.0                  dplyr_1.2.1
##  [99] codetools_0.2-20           BiocSingular_1.28.0
## [101] plotly_4.12.1              mime_0.13
## [103] splines_4.6.0              Rcpp_1.1.2
## [105] fastDummies_1.7.6          dbplyr_2.6.0
## [107] leaps_3.2                  RcppAlgos_2.10.1
## [109] gridtext_0.1.6             knitr_1.51
## [111] blob_1.3.0                 BiocVersion_3.23.1
## [113] lme4_2.0-6                 fs_2.1.0
## [115] listenv_1.0.0              checkmate_2.3.4
## [117] Rdpack_2.6.6               pkgbuild_1.4.8
## [119] ggsignif_0.6.4             estimability_2.0.0
## [121] tibble_3.3.1               Matrix_1.7-5
## [123] statmod_1.5.2              pkgconfig_2.0.3
## [125] tools_4.6.0                cachem_1.1.0
## [127] rbibutils_2.4.1            RSQLite_3.53.3
## [129] viridisLite_0.4.3          DBI_1.3.0
## [131] fastmap_1.2.0              rmarkdown_2.31
## [133] scales_1.4.0               grid_4.6.0
## [135] ica_1.0-3                  Seurat_5.5.1
## [137] broom_1.0.13               AnnotationHub_4.2.2
## [139] patchwork_1.3.2            BiocManager_1.30.27
## [141] dotCall64_1.2              carData_3.0-6
## [143] RANN_2.6.2                 rpart_4.1.27
## [145] snow_0.4-4                 farver_2.1.2
## [147] reformulas_0.4.4           yaml_2.3.12
## [149] MatrixGenerics_1.24.0      cli_3.6.6
## [151] purrr_1.2.2                lifecycle_1.0.5
## [153] rsconnect_1.10.1           uwot_0.2.4
## [155] mvtnorm_1.4-2              bluster_1.22.0
## [157] sessioninfo_1.2.4          backports_1.5.1
## [159] BiocParallel_1.46.0        gtable_0.3.6
## [161] ggridges_0.5.7             progressr_1.0.0
## [163] parallel_4.6.0             limma_3.68.5
## [165] jsonlite_2.0.0             edgeR_4.10.3
## [167] RcppHNSW_0.7.0             mitml_0.4-5
## [169] ggplot2_4.0.3              multcompView_0.1-12
## [171] bit64_4.8.4                brio_1.1.5
## [173] Rtsne_0.17                 spatstat.utils_3.2-4
```

```
## [175] BiocNeighbors_2.6.0         SeuratObject_5.4.0
## [177] mice_3.19.0                 metapod_1.20.0
## [179] dqrng_0.4.1                 spatstat.univar_3.2-0
## [181] shiny_1.14.0                htmltools_0.5.9
## [183] sctransform_0.4.3           rappdirs_0.3.4
## [185] glue_1.8.1                  spam_2.11-4
## [187] httr2_1.3.0                 XVector_0.52.0
## [189] rprojroot_2.1.1             scran_1.40.0
## [191] gridExtra_2.3.1             boot_1.3-32
## [193] flashClust_1.1-4            igraph_2.3.3
## [195] R6_2.6.1                    asmbPLS_1.0.0
## [197] tidyr_1.3.2                 SingleCellExperiment_1.34.0
## [199] cluster_2.1.8.2             pkgload_1.5.3
## [201] nloptr_2.2.1                DelayedArray_0.38.2
## [203] tidyselect_1.2.1            xml2_1.6.0
## [205] car_3.1-5                   future_1.75.0
## [207] rsvd_1.0.5                  KernSmooth_2.23-26
## [209] S7_0.2.2                    data.table_1.18.4
## [211] htmlwidgets_1.6.4           RColorBrewer_1.1-3
## [213] biomaRt_2.68.0              rlang_1.3.0
## [215] spatstat.sparse_3.2-0       spatstat.explore_3.8-2
```
